# Novel Classification of Cardiovascular Disease Subtypes Reveals Associations Between Mortality and Polyunsaturated Fatty Acids: Insights from the United Kingdom Biobank Study

**DOI:** 10.1016/j.cdnut.2024.104434

**Published:** 2024-08-05

**Authors:** Jiamei Li, Haiqing Zheng, Xuanhui Chen, Shuo Ma, Qing Li, Jiaqi Sun, Ziying Chen, Li Yunyi, Li Dantong, Lin Miao, Huiying Liang, Huixian Li

**Affiliations:** 1 Department of Epidemiology, School of Public Health, China Medical University, Shenyang, China; 2 Medical Big Data Center, Guangdong Provincial People’s Hospital (Guangdong Academy of Medical Sciences), Southern Medical University, Guangzhou, Guangdong Province, 510080, China; 3 Guangdong Provincial Key Laboratory of Artificial Intelligence in Medical Image Analysis and Application, Guangzhou, China; 4 Clinical Data Center, Guangzhou Women and Children's Medical Center, Guangzhou Medical University, Guangzhou, China; 5 Department of Epidemiology, School of Public Health, Southern Medical University, Guangzhou, China; 6 School of Software, South China University of Technology, China; 7 Guangdong Cardiovascular Institute, Guangdong Provincial People's Hospital, Guangdong Academy of Medical Sciences, Guangzhou, Guangdong Province, 510080, China

**Keywords:** polyunsaturated fatty acids, ω-3, ω-6, ω-6/ω-3 ratio, cardiovascular disease, subtypes, mortality

## Abstract

**Background:**

Traditional association studies of cardiovascular disease (CVD) categorizations and polyunsaturated fatty acids (PUFAs) yielded conflicting findings. We propose a novel classification system based on fundamental characteristics of cardiovascular patients, such as age, body mass index, waist–hip ratio, to more accurately assess the impact of PUFAs (plasma measures) such as omega (ω)-3 (n–3) and ω-6 on mortality in cardiovascular patients.

**Methods:**

Principal component analysis and *k*-means clustering were used to determine the CVD subtype. Variables included age, body mass index, waist–hip ratio, diastolic blood pressure, systolic blood pressure, total cholesterol, total triglycerides, high-density lipoprotein-cholesterol, apolipoprotein B:apolipoprotein A1, glycated hemoglobin, creatinine, albumin, C-reactive protein, white blood cell count, platelet count, and hemoglobin concentration. The association of PUFAs with all-cause, cardiovascular, and ischemic heart disease (IHD) mortality in patients with CVD was prospectively evaluated using restricted cubic splines and Cox proportional risk models.

**Results:**

Among the 35,096 participants, 3,786 fatalities occurred. Three distinct CVD subtypes were identified, with cluster 3 characterized by older age, male gender, and low high-density lipoprotein-cholesterol, having the highest risk of mortality. Clusters 2 and 3 had the highest DHA and ω-6/ω-3 ratios, respectively, compared with Cluster 1. The protective effects of total PUFAs, ω-3, and DHA were mainly reflected in all-cause mortality and were more significant in clusters 2 and 3. Furthermore, the ω-6/ω-3 ratio of the highest quartile increased risk of all-cause [Q3: hazard ratio (HR): 1.14, 95% confidence interval [CI]: 1.00, 1.29; Q4: HR: 1.41, 95% CI: 1.24, 1.61], CVD (Q4: HR: 1.36, 95% CI: 1.07, 1.75), and IHD mortality (Q4: HR: 1.17, 95% CI: 1.12, 2.03) in cluster 3 compared with the first quartile.

**Conclusions:**

Our findings highlight the heterogeneity of associations observed for the same type of PUFAs across distinct clusters. This association may be elucidated by the intricate interplay of various factors, encompassing inflammation, lipid metabolism, and cardiovascular health.

## Introduction

According to epidemiological studies, cardiovascular disease (CVD) continues to pose a major challenge to global health. The incidence and mortality of CVD have been steadily increasing globally [1,2]. CVD is a great threat to human life and health, which is characterized by high morbidity, high disability rate, and high mortality rate. Scientific evidence highlights PUFA deficiency as a risk factor for CVD mortality, especially deficiency of omega (ω)-3 and ω-6 [3,4]. However, the existing literature presents mixed findings regarding the effect of PUFAs on cardiovascular patient outcomes [[5], [6], [7]].

In addition, the assessment of dietary PUFA intake is challenging. Subjective assessments of PUFAs using food frequency questionnaires often introduce biases that may distort the relationship between PUFAs and disease outcomes. For example, one study showed that PUFA intake was not associated with PUFA’s serum concentration. Serum concentrations of PUFAs were effective in reducing all-cause mortality, whereas dietary intake of PUFAs was not effective in preventing mortality [7]. The absorption rate of PUFAs also varies greatly among different populations [7,8]. Thus, circulating PUFA concentrations serve as an objective biomarker of PUFA intake, thereby mitigating the inherent bias associated with self-reported dietary assessments [[9], [10], [11], [12]].

In recent years, artificial intelligence and machine learning (ML) have been widely used in health-related research, including predicting the association between circulating vitamin exposure and mortality risk [13]. Clustering is an unsupervised ML task that automatically discovers natural groupings in data, which may help identify underlying patterns in patients compared with simple stratification. In the current CVD research, studies have traditionally classified patients according to standard diagnoses, such as ischemic heart disease (IHD), stroke, and so on. Classifying patients with CVD according to basic characteristics such as patient's age, BMI, waist–hip ratio (WHR) has the potential to determine which group of cardiovascular patients will benefit from PUFAs and which group may not be suitable for this intervention. This approach holds promise for early prevention and reduced risk of death in patients with CVD.

In this study, we constructed a subtype model of cardiovascular patients using the United Kingdom Biobank database. We aimed to elucidate the association between different types of circulating PUFA concentrations and risk of all-cause and cause-specific mortality in patients with different subtypes of CVD. These efforts are designed to advance our understanding of the multifaceted relationship between circulating PUFAs, different CVD subtypes, and patient outcomes.

## Methods

### Study design

The study represents a robust prospective cohort analysis using the extensive and comprehensive United Kingdom Biobank database. The database successfully recruited a vast cohort of >500,000 participants aged between 37 and 73 y. These individuals were drawn from the general population and underwent assessments at 22 centers across England, Wales, and Scotland. The data collection phase took place between 2006 and 2010. Detailed information regarding the study design, the characteristics of the participants, and the stringent quality control measures employed in data acquisition have been comprehensively outlined in prior publications, ensuring the transparency and rigor of the study [14,15]. The United Kingdom Biobank received ethical approval from the Research Ethics Committee (REC reference for the UK Biobank is 11/NW/0382). The specific projects undertaken in this work were conducted using the United Kingdom Biobank data from project number 88365.

**FIGURE 1:**
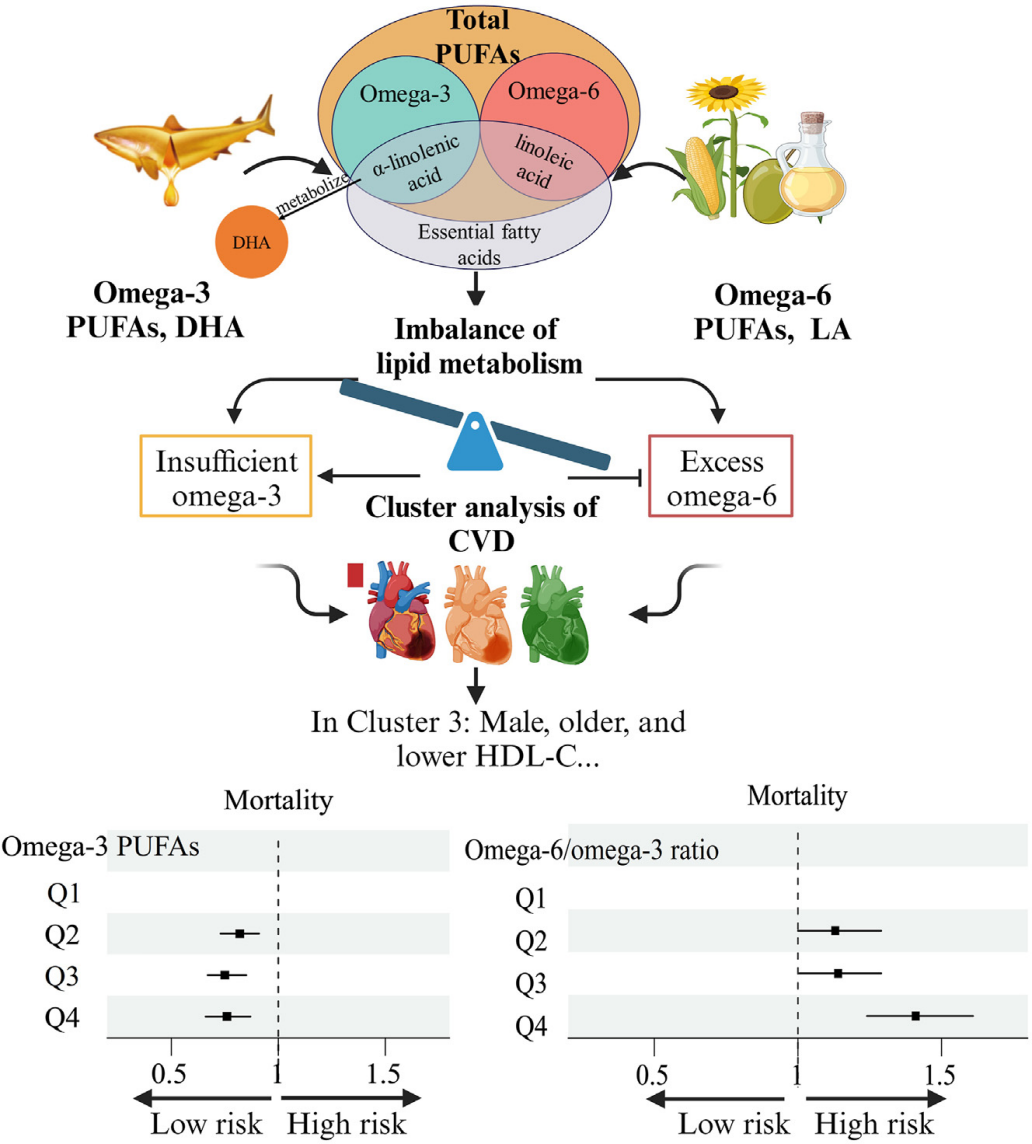
Graphical Abstract. The research findings distinctly indicate that higher levels of circulating ω-3 are associated with a lower risk of adverse outcomes, underscoring the positive role of ω-3 in cardiovascular health. However, the adverse outcomes of circulating ω-6 were not related to death outcomes in either cluster 1, cluster 2, or cluster 3 populations. However, how high the ω-6/ω-3 ratio would further affect the adverse outcomes in patients with cardiovascular disease remains unknown. CVD, cardiovascular disease; LA, linoleic acid.

This study unfolds in 2 distinct phases, each with a unique focus and set of objectives. In the first phase, the research design was tailored to unearth subtypes among patients with CVD using sophisticated cluster analysis methods. The central aim of this phase was to identify and characterize different subgroups of patients with CVD. This step is pivotal in shedding light on the heterogeneity within the broader population of patients with CVD, personalized health care strategies, and tailored interventions. The second phase of this study builds upon the insights gained in phase 1. Here, the research delves deeply into the effect of circulating PUFAs on the prognosis of individuals belonging to the various CVD subtypes identified earlier. This phase aimed to elucidate the effects of different circulating PUFA concentrations on the prognosis of different CVD subtypes. This approach is integral in advancing our understanding of the interaction between dietary factors, such as PUFAs, and the unique clinical profiles of patients with CVD. Examining these relationships within the context of different CVD subtypes provides a nuanced perspective that can pave the way for more targeted and effective therapeutic approaches in cardiovascular health. Please refer to Figure 1 for the graphic summary.

### Population

Participants in the United Kingdom Biobank completed a touch screen questionnaire, face-to-face interviews, a battery of physical measurements, and the provision of crucial biological samples. To ensure the relevance and specificity of the study, the inclusion criteria were tailored to target individuals with CVD. These participants met the eligibility criteria for CVD. They furnished an array of vital data by completing detailed questionnaires, attending face-to-face interviews, and providing physical measurements and biological samples. This robust data collection process allowed for a thorough exploration of the relationships between PUFAs and outcomes in patients with CVD. Conversely, the exclusion criteria refined the study population and minimized confounding factors. The excluded individuals fell into 2 main categories: *1)* individuals with a history of cancer and *2)* incomplete information concerning circulating PUFAs. The exclusion of patients with cancer was a logical choice, as cancer can introduce substantial variability in health outcomes and potentially obscure the specific effects of circulating PUFAs on CVD prognosis. Furthermore, the availability of complete and comprehensive data on circulating PUFAs was essential to maintain the integrity and scientific rigor of the analysis.

**FIGURE 2:**
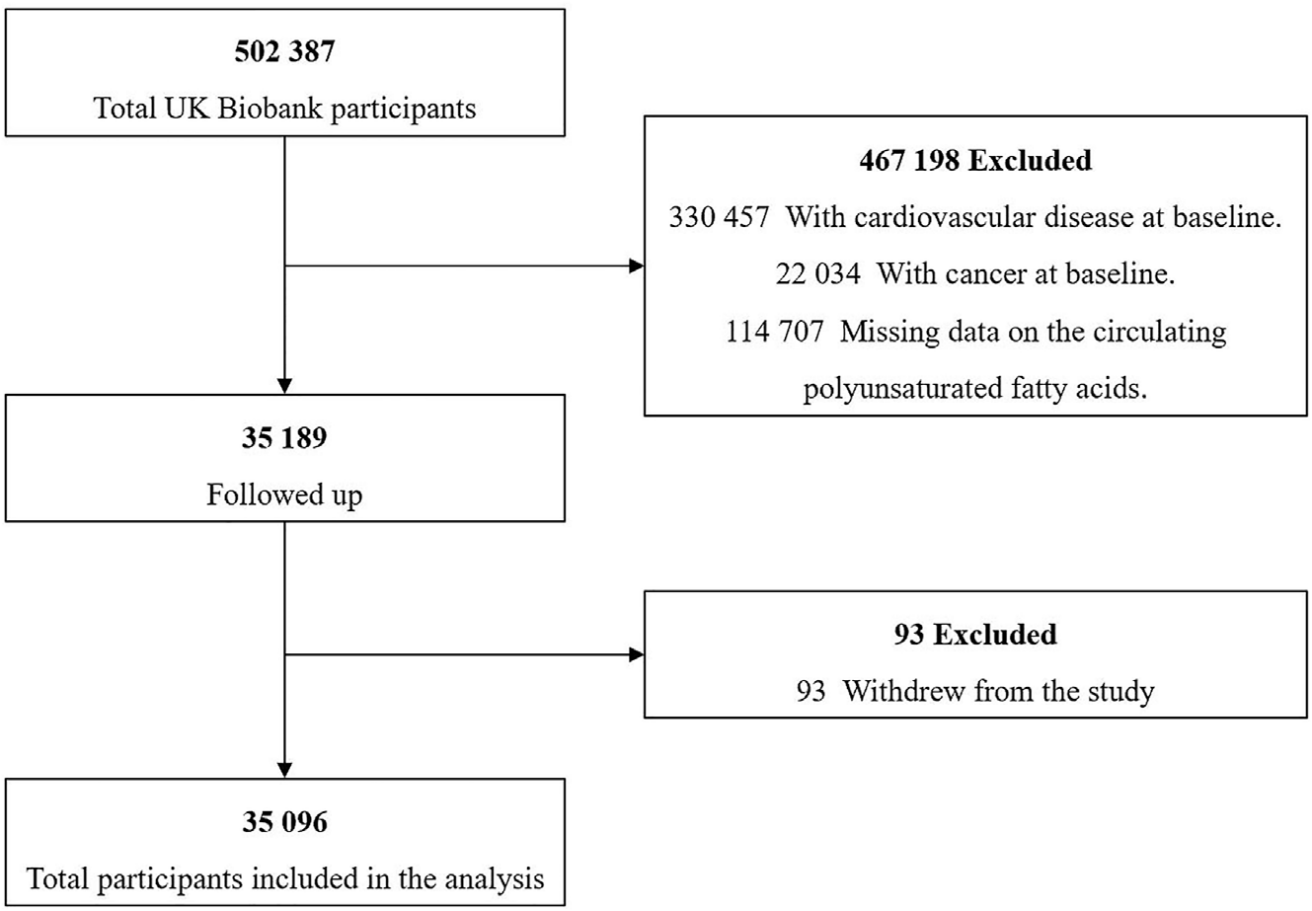
Flowchart of participant enrolment.

The analysis in our present study was based on a data set comprising 35,096 individuals. The details of the participant enrolment are shown in Figure 2.

### Measurements

#### Selection of clinical variables

**FIGURE 3:**
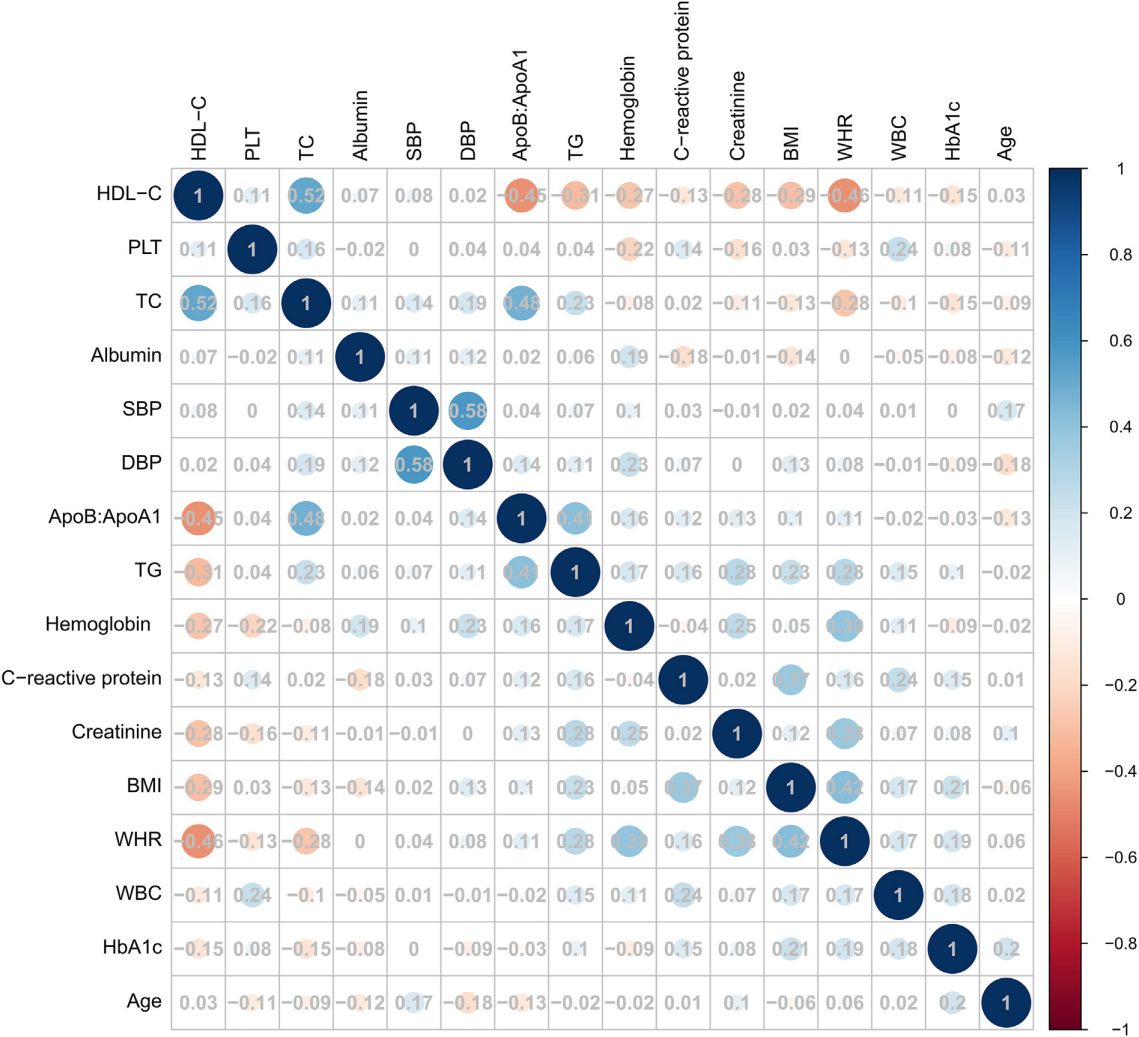
Linear correlation between all pairs of features. Highly correlated features were removed, with a threshold of 0.60, removing one of each pair of correlated features (Pearson correlation). ApoB:ApoA1, apolipoprotein B:apolipoproteinA1; DBP, diastolic blood pressure; HbA1c, glycated hemoglobin; PLT, platelet count; SBP, systolic blood pressure; TC, total cholesterol; TG, total triglycerides; WBC, white blood cell count; WHR, waist–hip ratio.

Twenty-one variables were initially selected to distinguish between different subtypes of patients with CVD. Those variables with >25% of values missing were dropped from the analysis to restrict the model to ensure the robustness of the data set and maintain the validity of subsequent statistical analyses. Supplemental Table 1 provides details of the missing covariates’ proportions. A cluster analysis of 18 variables was carried out. Highly correlated features were removed, with a threshold of 0.60, removing one of each pair of correlated features (Pearson correlation) [16]. Continuous features were standardized by converting each data set to have a mean of 0 and a standard deviation of 1, calculated by subtracting the mean from the specific value and dividing by the standard deviation. The 16 variables selected for clustering, including age, BMI, waist–hip ratio (WHR), diastolic blood pressure (DBP), systolic blood pressure (SBP), total cholesterol (TC), total triglycerides (TG), HDL-cholesterol, apolipoprotein B:apolipoproteinA1 (ApoB:ApoA1), glycated hemoglobin (HbA1c), creatinine, albumin, C-reactive protein, white blood cell count (WBC), platelet count (PLT), and hemoglobin concentration. A heatmap showing pairwise correlations among the cluster variables is presented in Figure 3 and Supplemental Figure 1.

#### Exposure assessment

Circulating PUFA concentrations in blood samples collected between 2006 and 2010 were measured by a targeted high-throughput nuclear magnetic resonance, including total PUFAs, ω-3, DHA, ω-6, linoleic acid (LA), and the ω-6/ω-3 ratio.

### Definitions of CVD

Patients with CVD were identified as individuals who either had a confirmed physician diagnosis of CVD at baseline or whose hospital inpatient data indicated a CVD diagnosis before their enrolment. The criteria for defining CVD were based on the International Classification of Diseases, 10th revision (ICD-10): CVD (G45, I10-I15, I20-I25, I42, I48, I50, I60-I64, I69, I73 and I80), as well as IHD (I20-I25) [17,18].

### Outcomes

The study's primary outcomes were all-cause mortality and secondary outcomes were CVD and IHD mortality. Mortality data for participants were obtained from NHS England for those residing in England and Wales, and from the NHS Central Register, which is a part of the National Records of Scotland, for participants in Scotland. Data collation showed that the information on deaths in England and Wales was available up to October 31, 2021, and in Scotland, it extended to November 12, 2021. Consequently, these dates were established as the respective follow-up cutoff times for England/Wales and Scotland unless death occurred.

### Covariates

Baseline questionnaires reported by the participants were used to assess various potentially confounding variables: *1)* sociodemographic factors, including gender and household income (less than £18,000, £18,000 to £30,999, £31,000 to £51,999, £52,000 to £100,000 and greater than £100,000); *2)* socioeconomic status, including Townsend deprivation index; *3)* lifestyle habits, including smoking status (never, former, and current), alcohol status (never, former, and current), and physical activity (low, moderate, and high); *4)* comorbidities, including hypertension and diabetes; *5)* drug use, including cholesterol-lowering medication use, antihypertensive drugs use, insulin treatment, and aspirin use. Hypertension was defined as using antihypertensive drugs, a DBP of 90 mmHg or higher, a SBP of 140 mmHg or higher, or a self-reported history of hypertension. Diabetes was defined as the current use of hypoglycemic drugs or a self-reported history of diabetes.

### Statistical analysis

Outliers for each clustering were identified using the boxplot rule, which considers data points that fall outside ± 1.5 times the IQR. The box diagram of clustering indicators is shown in Supplemental Figure 2. Outliers were subsequently removed from the analysis to prevent them from unduly influencing the analysis. Multiple imputation with chained equations was employed to account for any missing variables. The distribution patterns of clustering variables were evaluated by creating Q–Q plots in Supplemental Figure 3. Notably, a clear right-skewed distribution pattern was observed for C-reactive protein, and specifically, this variable was log-transformed to approximate a normal distribution. Before clustering, all variables were standardized.

**FIGURE 4:**
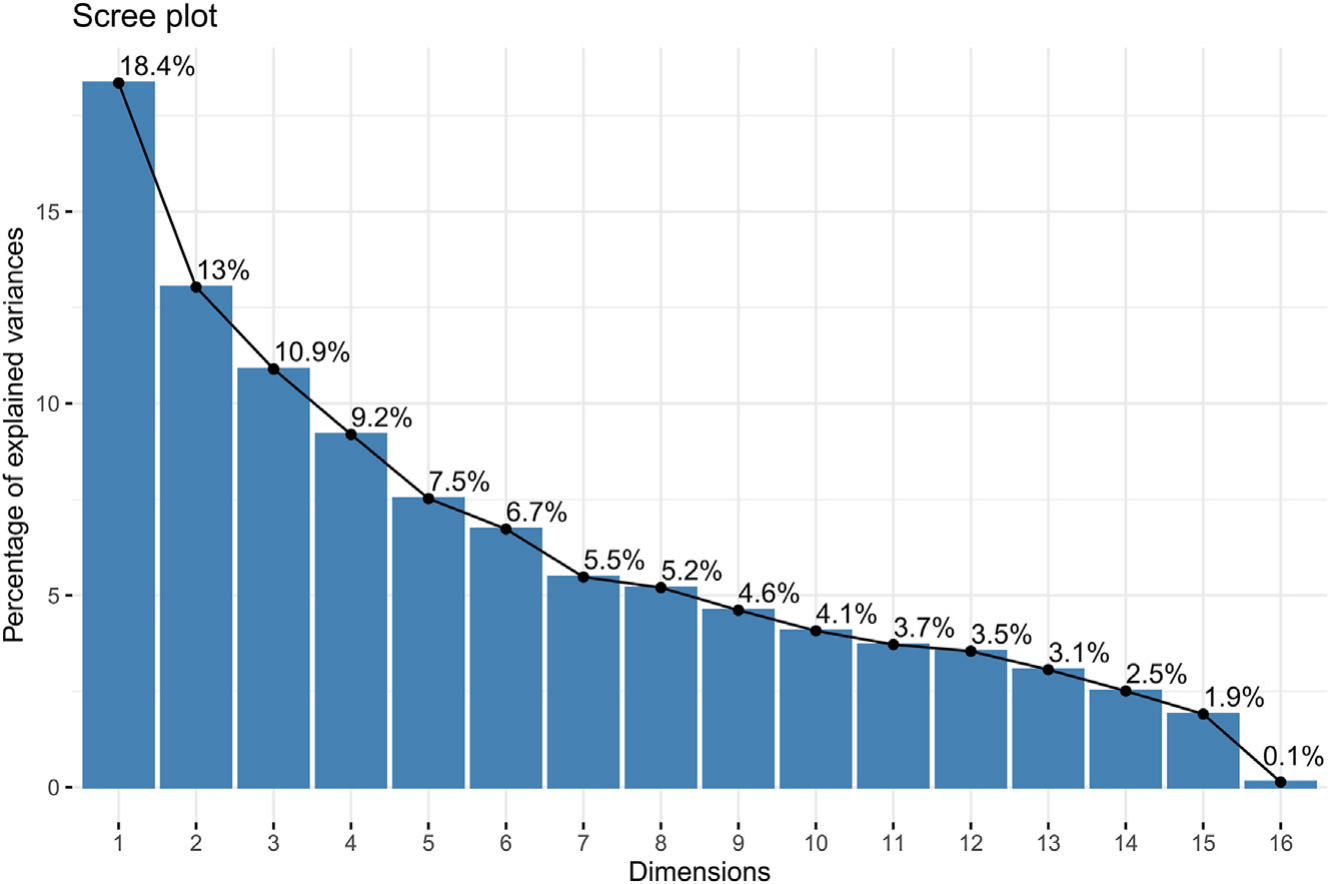
Scree plot of the proportion of variance in the data explained by each principal component.

**FIGURE 5:**
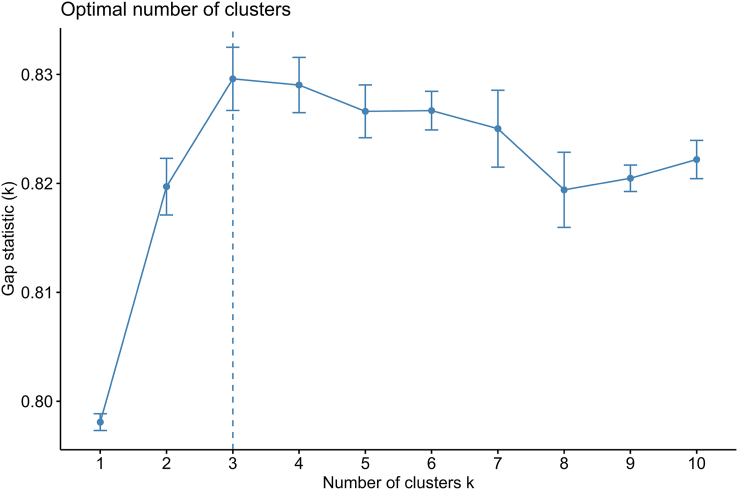
The gap statistics for the *k*-means have determined the optimal number of clusters. *K* = 3 is the optimal number of clusters.

**FIGURE 6:**
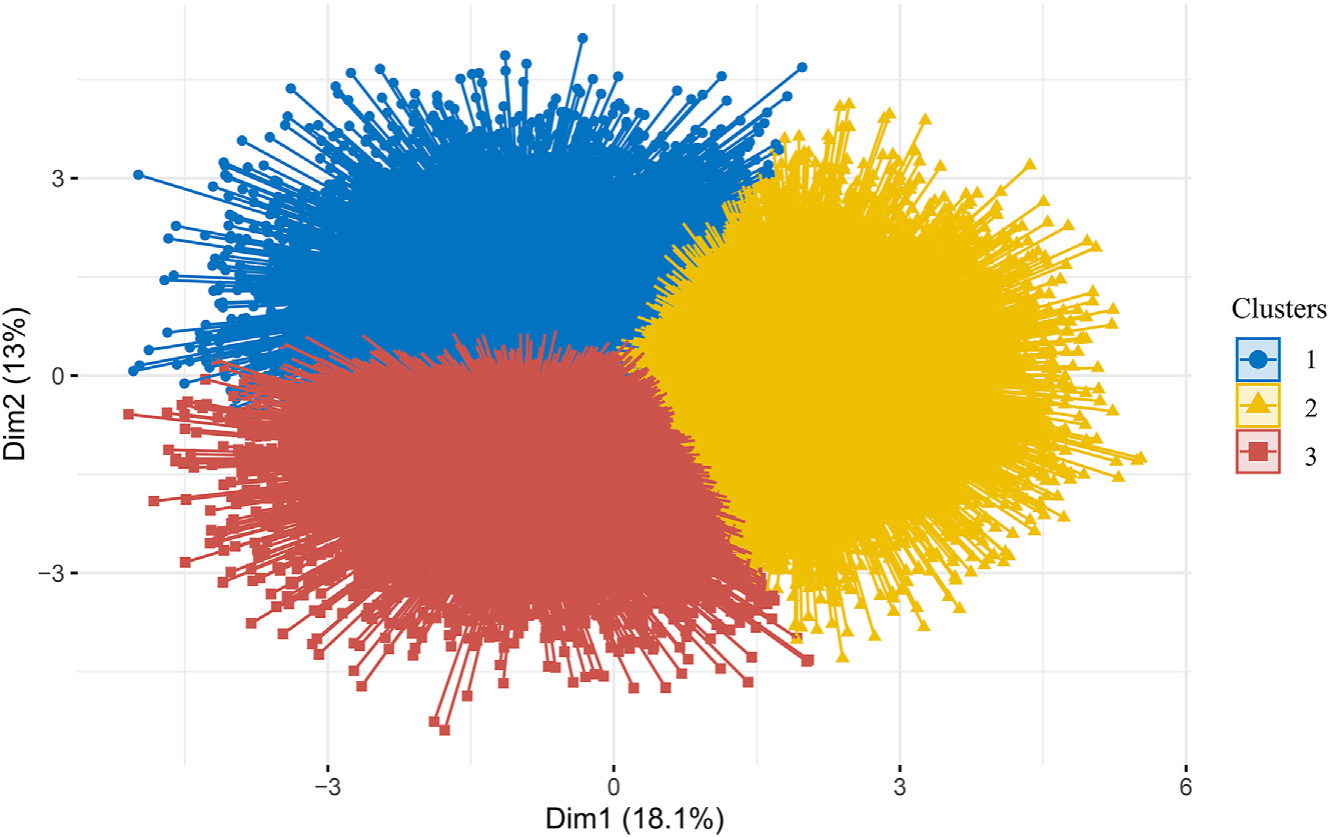
The visualization of the clustering data in the 2D space.

Principal component analysis was applied to reduce the dimensionality of the data set. Specifically, the number of principal components (PCs) that collectively explained ≥ 80% of the variance in the data was selected. In this study, 9 PCs were chosen as inputs for the clustering model. A scree plot of the proportion of variance explained by each PC is shown in Figure 4. The 2-step clustering method was used for clustering. In the first step, it estimated the optimal number of clusters based on gap statistics, and in the second step, *k*-means clustering was applied. Gap Statistic is a statistical method used to determine the number of clusters, which evaluates the quality of cluster results by comparing the total internal variation of a cluster with that of a reference data set. Ideally, the number of clusters chosen should have the largest Gap Statistic and be significantly different from the Gap Statistic corresponding to the next cluster number. The optimal number of clusters is shown in Figure 5, with the 3-cluster (*k* = 3) model adopted in this study. The clustering results are visualized in Figure 6.

In the description of baseline characteristics, the Q–Q diagram was first used to test the normality of continuous variables. The distribution patterns of continuous variables were evaluated by creating Q–Q plots in Supplemental Figure 4. If the continuous variables were normally distributed, the mean and standard deviation were used to represent them; otherwise, the median and its IQR were used to represent them. The categorical variables are represented by numbers (%). Then, to visualize the distribution of PUFAs (total PUFAs, ω-3, DHA, ω-6, LA, and the ω-6/ω-3 ratio) in different clusters and facilitate group comparisons, violin plots were employed. Between-group comparisons for categorical variables were conducted using the chi-square test, whereas the Kruskal–Wallis test was used for continuous variables. Dunn's test was used for pairwise comparison after the difference between multiple groups was statistically significant. The Kaplan–Meier method was employed to explore outcomes for different cluster groups. Exposure variables were divided into 4 groups based on quartiles (<P25, P25-P50, P50-P75, ≥P75). This methodology ensures a more balanced sample size across the groups, thereby mitigating the potential bias associated with uneven grouping. Additionally, it facilitates the evaluation of potential nonlinear effects. The multivariate restricted cubic spline models (RCS) were used to evaluate the relationships between PUFAs (total PUFAs, ω-3, DHA, ω-6, LA, and the ω-6/ω-3 ratio) and all-cause, CVD, and IHD mortality. Multivariate Cox regression models were used to assess the association of PUFAs with outcomes in different clusters. The RCS models and Cox regression models were adjusted for various covariates to reduce confounding bias, including age, gender, Townsend deprivation index, household income, physical activity, smoking status, alcohol status, cholesterol-lowering medication use, antihypertensive drugs use, insulin treatment, aspirin use, diabetes, and hypertension. Subgroup analysis of PUFAs (total PUFAs, ω-3, DHA, ω-6, LA, and the ω-6/ω-3 ratio) on all-cause, CVD, and IHD mortality was performed for gender (male, female), age (<60, ≥60 y), history of diabetes (no, yes), and history of hypertension (no, yes). Subgroup analysis models were adjusted for gender, age, Townsend deprivation index, household income, physical activity, smoking status, alcohol status, cholesterol-lowering medication use, antihypertensive drugs use, insulin treatment, aspirin use, diabetes, and hypertension.

#### Power analysis

For the survival and onset analyses, we utilized the powerCT function within the R package powerSurvEpi (version 0.1.0), setting the *P* value threshold at 0.05, to calculate the statistical power.

All statistical analyses, including gap statistics and *k*-means clustering, were conducted using R version 4.3.0, and a significance level of 0.05 (2-sided) was used to determine statistical significance. The Benjamini–Hochberg method was used to calibrate the Cox and the RCS model for multiple tests.

## Results

### Baseline characteristics of all participants

**FIGURE 7:**
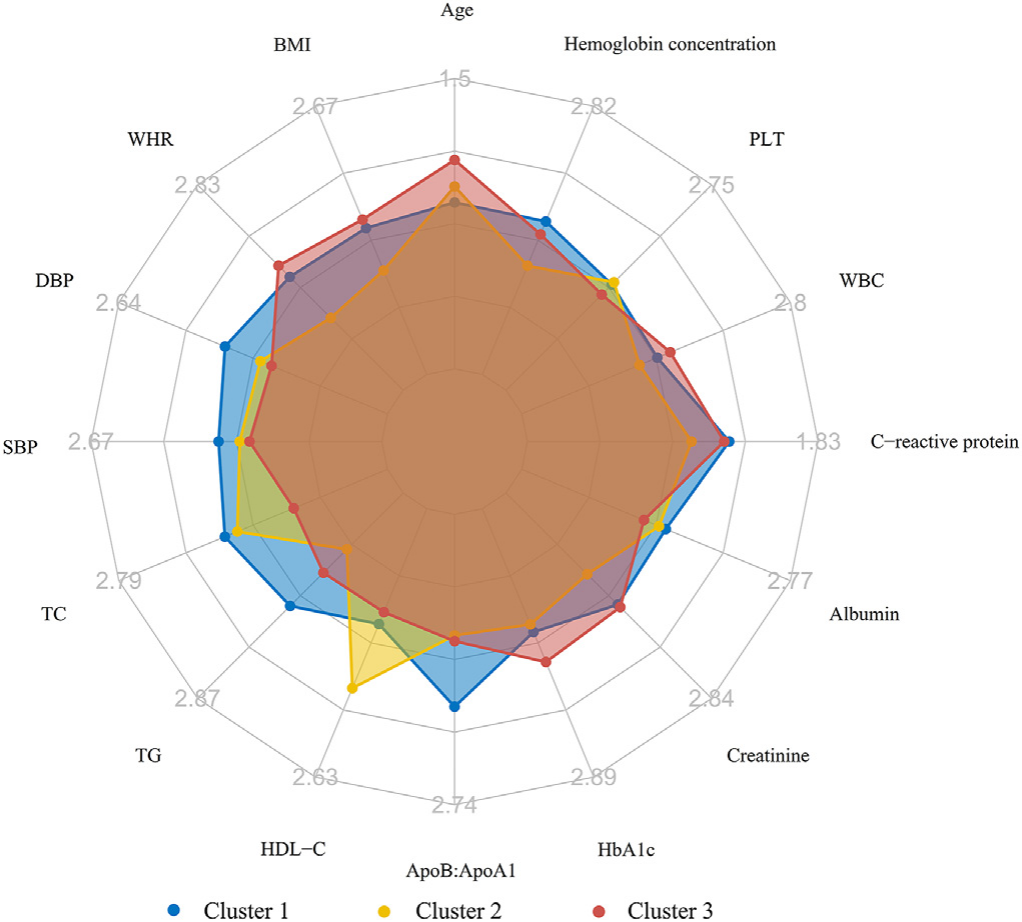
Profiles of the 3 Clusters from the prospective cohort. Radar plots were drawn for each cluster using z-values, which were calculated by adjusting the cluster mean for each variable to the cohort mean and SD for each variable. We then compared the radar plots visually and described the particular characteristics of each cluster. ApoB:ApoA1, apolipoprotein B:apolipoproteinA1; DBP, diastolic blood pressure; HbA1c, glycated hemoglobin; PLT, platelet count; SBP, systolic blood pressure; SD, standard deviation; TC, total cholesterol; TG, total triglycerides; WBC, white blood cell count; WHR, waist–hip ratio.

Table 1 shows the baseline characteristics of study participants categorized by clusters. The study included a total of 35,096 individuals (median [Q1, Q3] age, 61.0 [55.0, 65.0] y; 46.7% female). Throughout the follow-up period, a total of 3,786 people passed away, with 941 and 600 deaths attributed to CVD and IHD, respectively. To highlight the significant differences between clusters, the radar plot was used to characterize the *z*-score distribution of each cluster variable in each cluster, as shown in Figure 7. Cluster 1 consisted of the youngest individuals with elevated levels of ApoB:ApoA1, TC, TG, SBP, and DBP. Cluster 2 predominantly comprised females (77.9%) with the lowest BMI, WHR, TG, creatinine, C-reactive protein, WBC, and hemoglobin concentration, while having the highest levels of HDL-cholesterol. Cluster 3 primarily consisted of males (72.7%) who were older, with a higher BMI, WHR, and HbA1c, and a lower TC, HDL-cholesterol, and PLT.

**TABLE 1:** Baseline characteristics of the study participants by clusters^1^.

Clinical variables	Overall (*n* = 35,096)	Cluster 1 (*n* = 11,746)	Cluster 2 (*n* = 11,543)	Cluster 3 (*n* = 11,807)	*K − W/χ*^*2*^	*P* value
Age, median [Q1, Q3], y	61.0 [55.0, 65.0]	58.0 [52.0, 63.0]	61.0 [54.0, 65.0]	63.0 [59.0, 66.0]	2348.01	<0.001
Female, *n* (%)	16,391 (46.7)	4179 (35.6)	8988 (77.9)	3224 (27.3)	6872.06	<0.001
BMI, median [Q1, Q3], kg/m^2^	28.4 [25.6, 31.7]	29.3 [26.9, 32.5]	25.6 [23.5, 28.1]	30.1 [27.4, 33.6]	7363.46	<0.001
Townsend deprivation index, median [Q1, Q3]	−2.0 [−3.6, 1.0]	−2.1 [−3.6, 0.8]	−2.2 [−3.7, 0.5]	−1.7 [−3.4, 1.6]	188.78	<0.001
Household income, *n* (%), £					649.95	<0.001
<18,000	11,265 (32.1)	3195 (27.2)	3434 (29.7)	4636 (39.3)		
18,000–30,999	9283 (26.5)	2997 (25.5)	3032 (26.3)	3254 (27.6)		
31,000–51,999	7726 (22.0)	2920 (24.9)	2611 (22.6)	2195 (18.6)		
52,000–100,000	5376 (15.3)	2135 (18.2)	1890 (16.4)	1351 (11.4)		
>100,000	1446 (4.1)	499 (4.2)	576 (5.0)	371 (3.1)		
Physical activity, *n* (%)					402.59	<0.001
Low	7481 (21.3)	2579 (22.0)	1860 (16.1)	3042 (25.8)		
Moderate	14,314 (40.8)	4653 (39.6)	4789 (41.5)	4872 (41.3)		
High	13,301 (37.9)	4514 (38.4)	4894 (42.4)	3893 (33.0)		
Smoking status, *n* (%)					634.56	<0.001
Never	17,697 (50.4)	6061 (51.6)	6679 (57.9)	4957 (42.0)		
Former	13,822 (39.4)	4380 (37.3)	3988 (34.5)	5454 (46.2)		
Current	3577 (10.2)	1305 (11.1)	876 (7.6)	1396 (11.8)		
Alcohol status, *n* (%)					186.43	<0.001
Never	1798 (5.1)	489 (4.2)	567 (4.9)	742 (6.3)		
Former	1562 (4.5)	461 (3.9)	378 (3.3)	723 (6.1)		
Current	31,736 (90.4)	10,796 (91.9)	10,598 (91.8)	10,342 (87.6)		
Cholesterol-lowering medication use, n (%)	14,284 (40.7)	2407 (20.5)	3 359 (29.1)	8 518 (72.1)	7467.75	<0.001
Antihypertensive drugs use, *n* (%)	21,000 (59.8)	6119 (52.1)	5 883 (51.0)	8 998 (76.2)	1987.86	<0.001
Insulin treatment, *n* (%)	852 (2.4)	86 (0.7)	161 (1.4)	605 (5.1)	556.96	<0.001
Aspirin use, *n* (%)	10,908 (31.1)	2232 (19.0)	2668 (23.1)	6008 (50.9)	3303.92	<0.001
Diabetes, *n* (%)	4425 (12.6)	776 (6.6)	513 (4.4)	3136 (26.6)	3168.17	<0.001
Hypertension, *n* (%)	30,417 (86.7)	10,536 (89.7)	9363 (81.1)	10,518 (89.1)	461.10	<0.001

Abbreviation: BMI, body mass index.

1 Values are *n* (%) and median [IQR]. Between-group comparisons for categorical variables were conducted using the *χ* test, whereas the Kruskal–Wallis test was used for continuous variables. Significant effect of clinical variables and clusters (*P* value < 0.05).

### Comparisons of the distribution of PUFAs among the clusters

**FIGURE 8:**
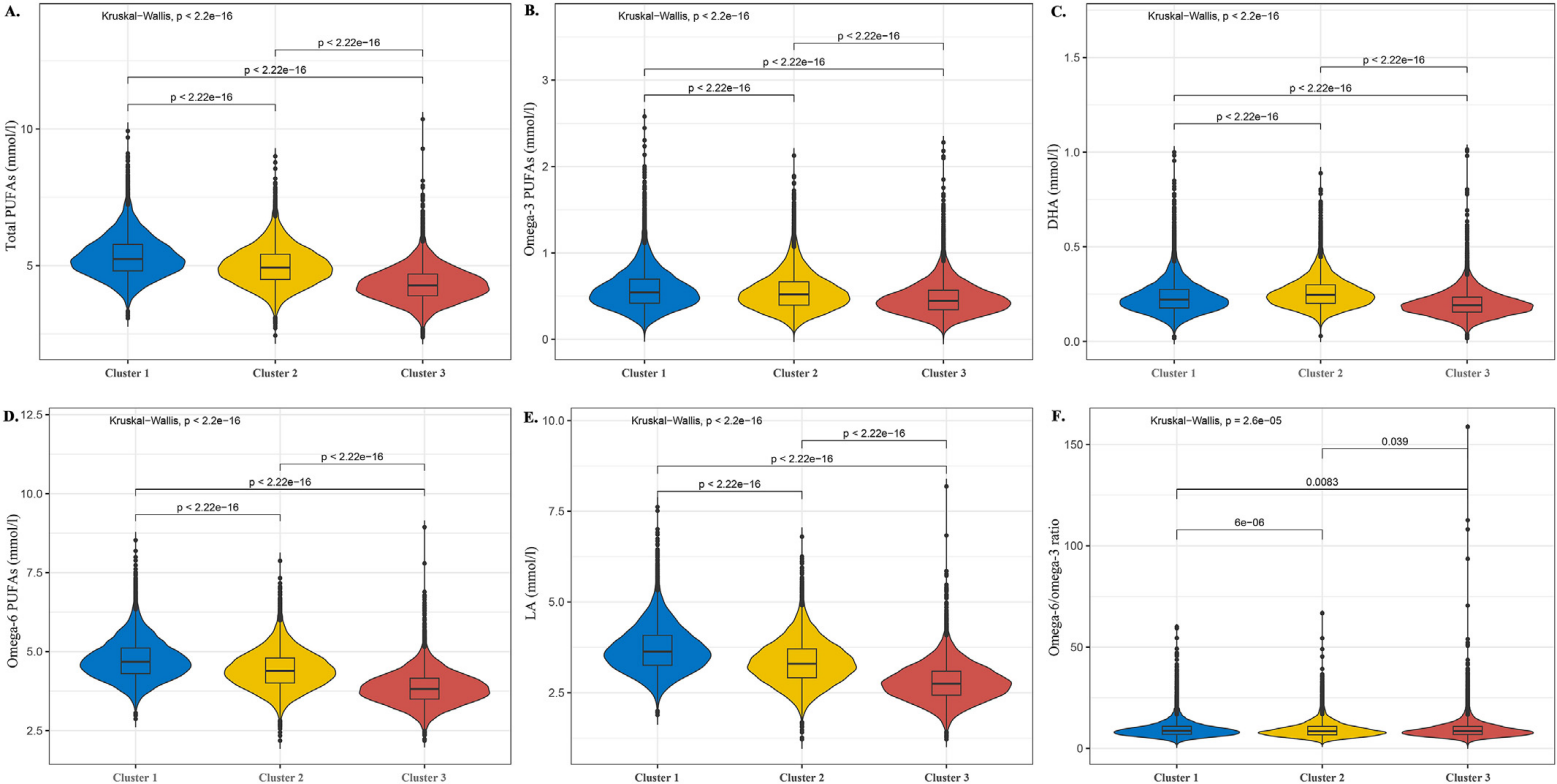
Distribution of PUFAs by clusters in participants. Kruskal–Wallis test for between-group differences in the distribution of PUFAs in different clusters. (A) Distribution of total PUFAs in different cluster groups. (B) Distribution of ω-3 in different cluster groups. (C) Distribution of DHA in different cluster groups. (D) Distribution of ω-6 in different cluster groups. (E) Distribution of LA in different cluster groups. (F) Distribution of the ω-6/ω-3 ratio in different cluster groups. LA, linoleic acid.

Significant differences existed in the levels of various PUFAs among the 3 clusters. Cluster 1 participants exhibited significantly higher levels of various PUFAs than those in clusters 2 and 3, these included total PUFAs [average levels: 5.25 (4.80, 5.77) vs. 4.93 (4.49, 5.41), 4.28 (3.90, 4.69)], ω-3 [average levels: 0.54 (0.42, 0.70) vs. 0.52 (0.40, 0.67), 0.45 (0.34, 0.57)], ω-6 [average levels: 4.68 (4.30, 5.12) vs. 4.39 (4.01, 4.80), 3.82 (3.50, 4.16)], and LA [average levels: 3.63 (3.25, 4.08) vs. 3.30 (2.91, 3.71), 2.75 (2.43, 3.09)]. In contrast, cluster 2 participants exhibited the highest DHA levels [average levels: 0.25 (0.20, 0.30) vs. 0.22 (0.18, 0.27), 0.19 (0.16, 0.23)] and the lowest ω-6/ω-3 ratios compared with clusters 1 and 3 [average levels: 8.46 (6.75, 10.85) vs. 8.70 (6.95, 10.91), 8.56 (6.90, 10.83)]. In cluster 3, to the contrary, had the highest ω-6/ω-3 ratio compared with the other clusters, whereas their levels of other PUFAs were generally lower. The distribution of PUFAs by clusters in participants is shown in Figure 8.

### Comparisons of risk of mortality among the clusters

**FIGURE 9:**
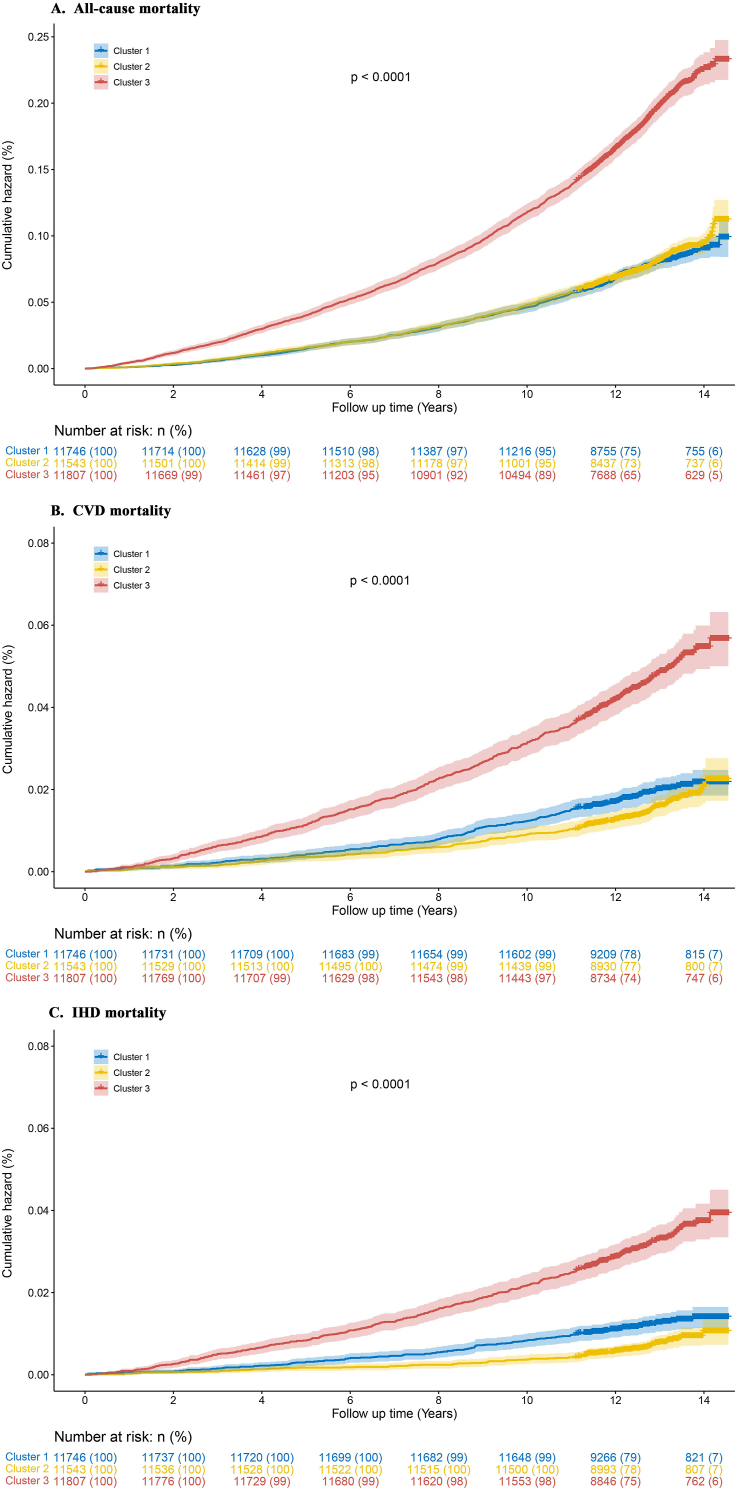
Cumulative mortality rates by clusters with the Kaplan–Meier method. (A) Cumulative risk of all-cause mortality by clusters. (B) Cumulative risk of CVD mortality by clusters. (C) Cumulative risk of IHD mortality by clusters. CVD, cardiovascular disease; IHD, ischemic heart disease.

We compared mortality risk for all-cause, CVD, and IHD mortality across 3 clusters. The findings, as illustrated in Figure 9 and Supplemental Table 2, revealed that cluster 3 patients had the highest risk for all-cause, CVD, and IHD mortality (Figure 9). There was no statistically significant difference in cumulative all-cause mortality between clusters 1 and 2. However, cluster 1 had slightly higher rates of CVD and IHD mortality than cluster 2 (*P* < 0.05) (Figure 9).

### Comparisons of associations of PUFAs and outcomes within the clusters

**FIGURE 10:**
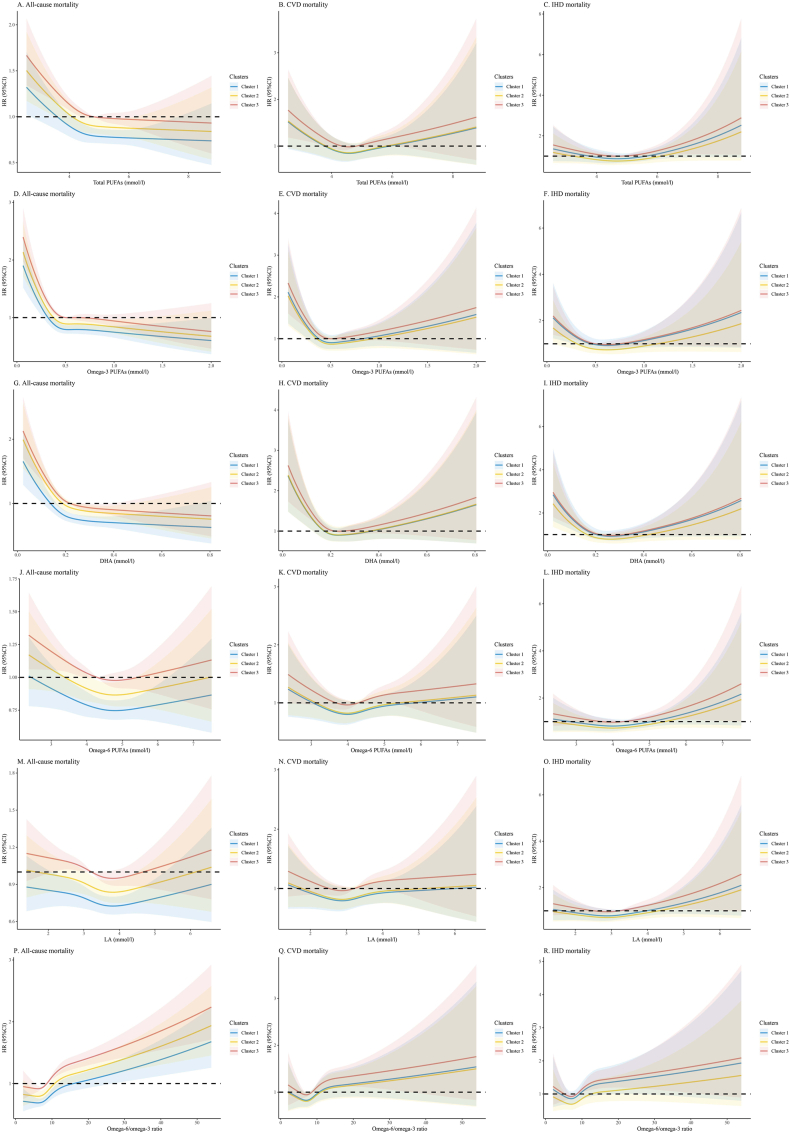
Restricted cubic spline models for the relationships between total PUFAs, ω-3, DHA, ω-6, LA, and the ω-6/ω-3 ratio with all-cause, CVD, and IHD mortality. (A) The relationships between total PUFAs and all-cause mortality. (B) The relationships between total PUFAs and cardiovascular disease mortality. (C) The relationships between total PUFAs and IHD mortality. (D) The relationships between ω-3 and all-cause mortality. (E) The relationships between ω-3 and CVD mortality. (F) The relationships between ω-3 and IHD mortality. (G) The relationships between DHA and all-cause mortality. (H) The relationships between DHA and CVD mortality. (I) The relationships between DHA and IHD mortality. (J) The relationships between ω-6 and all-cause mortality. (K) The relationships between ω-6 and cardiovascular disease mortality. (L) The relationships between ω-6 and IHD mortality. (M) The relationships between LA and all-cause mortality. (N) The relationships between LA and cardiovascular disease mortality. (O) The relationships between LA and IHD mortality. (P) The relationships between the ω-6/ω-3 ratio and all-cause mortality. (Q) The relationships between the ω-6/ω-3 ratio and cardiovascular disease mortality. (R) The relationships between the ω-6/ω-3 ratio and IHD mortality. Restricted cubic spline models are adjusted for gender, age, Townsend deprivation index, household income, physical activity, smoking status, alcohol status, cholesterol-lowering medication use, antihypertensive drugs use, insulin treatment, aspirin use, diabetes, and hypertension. CVD, cardiovascular disease; HR, hazard ratio; IHD, ischemic heart disease; LA, linoleic acid.

RCS models were used to examine the relationships between total PUFAs, ω-3, DHA, ω-6, LA, and the ω-6/ω-3 ratio with all-cause, CVD, and IHD mortality, as shown in Figure 10 and Supplemental Table 3.

In multivariate models, we found significant nonlinear relationships between total PUFAs, ω-3, and DHA and risk of all-cause, CVD, and IHD mortality (all *P* for nonlinearity < 0.05). There was an inverse L-shaped exposure–response relationship between these PUFAs and risk of all-cause mortality, whereas there was a U-shaped exposure–response relationship for risk of CVD and IHD mortality. In the absence of these PUFAs, risk of the outcome increases. Within a specific range, higher ω-3 and DHA concentrations are associated with a reduced risk of outcomes [hazard ratio (HR) < 1.0]. However, once PUFAs reach a specific threshold level, risk stabilizes and no longer continues to decrease or even increase. Cluster 1 displayed the lowest HR under similar exposure levels in all-cause mortality (Figure 10).

We found significant linear relationships between ω-6 and LA and risk of the studied outcomes (all *P* for linearity < 0.05). High level of these PUFAs were associated with low risk of all-cause and CVD mortality but were associated with an increased risk of IHD mortality. To the contrary , there was a J-shaped exposure–response relationship between ω-6/ω-3 ratio and risk of all-cause mortality, whereas there were only significant linear relationships for risk of CVD and IHD mortality (Figure 10). In general, a higher ω-6/ω-3 ratio leads to increased risk for all outcomes.

**FIGURE 11:**
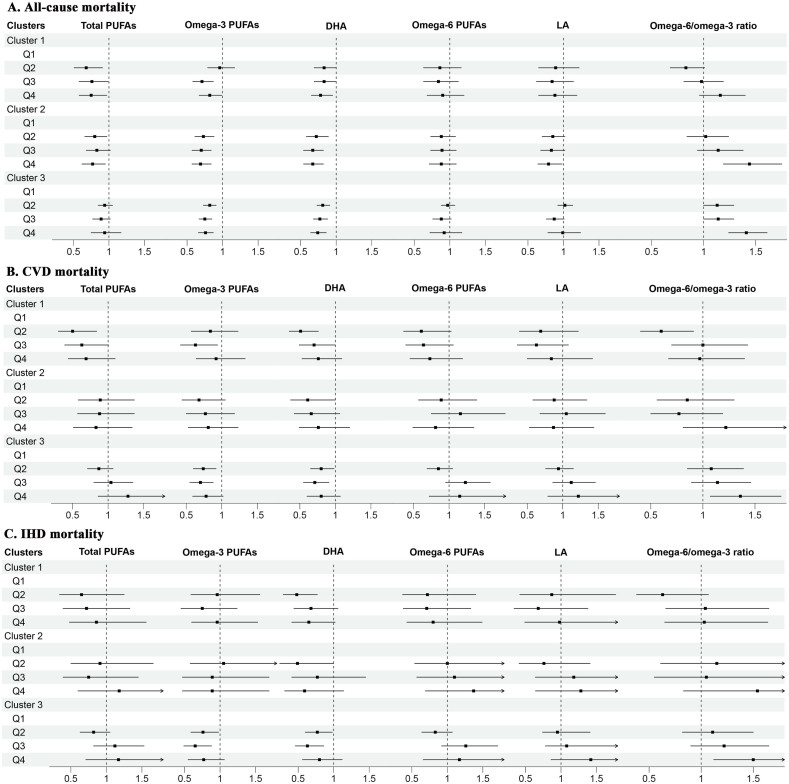
Association between PUFAs and all-cause, cardiovascular disease, and cardiovascular disease mortality. PUFAs variables were categorized into 4 groups (Q1, Q2, Q3, and Q4) by corresponding 25th, 50th, and 75th quartiles, and the Q1 groups were regarded as the reference (HR: 1). (A) Association between PUFAs and all-cause mortality. (B) Association between PUFAs and cardiovascular disease mortality. (C) Association between PUFAs and cardiovascular disease mortality. Adjusted for gender, age, Townsend deprivation index, household income, physical activity, smoking status, alcohol status, cholesterol-lowering medication use, antihypertensive drugs use, insulin treatment, aspirin use, diabetes, and hypertension. CVD, cardiovascular disease; IHD, ischemic heart disease.

To analyze the association between PUFAs with all-cause, CVD, and IHD mortality, we categorized PUFAs into 4 quartile groups (total PUFAs: <4.298, 4.298–4.816, 4.817–5.369, ≥5.370 mmol/L; ω-3: <0.383, 0.383–0.499, 0.500–0.645, ≥0.646 mmol/L; DHA: <0.175, 0.175–0.217, 0.218–0.271, ≥0.270 mmol/L; ω-6: <3.848, 3.848–4.290, 4.291–4.767, ≥4.768 mmol/L; LA: <2.775, 2.775–3.216, 3.217–3.699, ≥3.700 mmol/L; and a ω-6/ω-3 ratio: <6.879, 6.879–8.574, 8.575–10.682, ≥10.863). We observed distinct patterns for each cluster in the multivariable adjusted Cox model, as shown in Figure 11.

Within cluster 1, elevated concentrations of total PUFAs, ω-3, and DHA were significantly associated with a reduced risk of all-cause mortality and CVD mortality. To be specific, in comparison with the first quartile, the 3 remaining quartiles of total PUFAs were linked to lower risks of all-cause mortality (Q2 group: HR: 0.68, 95% confidence interval [CI]: 0.51, 0.91; Q3 group: HR: 0.76, 95% CI: 0.58, 0.99; Q4: HR: 0.75, 95% CI: 0.58, 0.97), and only the second quartile of total PUFAs were linked to lower risks of CVD mortality (HR: 0.50, 95% CI: 0.30, 0.84). The third quartile of ω-3 were linked to lower risks of all-cause (HR: 0.71, 95% CI: 0.58, 0.87) and CVD mortality (HR: 0.63, 95% CI: 0.42, 0.94), and the fourth quartile were linked to lower risks of all-cause mortality (HR: 0.82, 95% CI: 0.67, 0.99) than the first quartile. Moreover, the second quartile of DHA demonstrated a diminished risk of all-cause, CVD, and IHD mortality when compared to the first quartile (all-cause mortality: HR: 0.83, 95% CI: 0.69-1.00; CVD mortality: HR: 0.51, 95% CI: 0.35-0.76; IHD mortality: HR: 0.48, 95% CI: 0.29, 0.77). In cluster 1, a statistically significant lower risk of CVD mortality was observed for the second quartile of the ω-6/ω-3 ratio when compared with the first quartile (HR: 0.60, 95% CI: 0.40, 0.91), although this association was not evident at other ratio levels. Conversely, in this cluster, no significant associations were identified between ω-6 and LA with all-cause, CVD, and IHD mortality.

The relationship between cluster 2 and PUFAs mirrors that observed in cluster 1. Similarly, it displayed a lower risk of all-cause mortality in the second and fourth quartiles of total PUFAs than in the first quartile. In cluster 2, we also noted a significant reduction in risk of all-cause mortality across the 3 remaining quartiles of ω-3 and DHA compared with the first quartile. However, in this cluster, it is important to note that the ω-6/ω-3 ratio exhibited a significant increase in risk of all-cause mortality in the fourth quartile (HR: 1.44, 95% CI: 1.19, 1.75). Unlike cluster 1, in cluster 2, no significant associations were observed between ω-6 and LA with all-cause, CVD, and IHD mortality, except for the highest levels of LA, which acted as a protective factor against all-cause mortality (HR: 0.79, 95% CI: 0.64, 0.98).

Cluster 3 participants did not exhibit a significant association between total PUFAs and all-cause, CVD, and IHD mortality. Nevertheless, they displayed a reduced risk of all-cause mortality across all 3 quartiles of ω-3 and DHA when compared with the first quartile. Additionally, within cluster 3, there were noteworthy reductions in CVD and IHD mortality for the second and third quartiles of ω-3 and DHA, although such reductions were not evident in the fourth quartile. It is worth noting that, within this group, ω-6 and LA displayed negative associations with all-cause mortality, but paradoxically exhibited positive associations with CVD and IHD mortality, although these associations did not reach statistical significance. Moreover, it is important to highlight that a higher ω-6/ω-3 ratio significantly increased risk of all-cause, CVD, and IHD mortality, with this effect becoming statistically significant at levels exceeding the fourth quartile. Power analysis showed that our cohort had a 77% power to detect a 10% change in expected hazard for all-cause mortality, 29% for CVD mortality, and 21% for IHD mortality.

### Subgroup analysis

The results of subgroup analyses are presented in Supplemental Tables 4–6. Among cluster 1 participants, using the first quartile as the reference group, the protective effect of total PUFAs on risk of all-cause mortality was found in the third and fourth quartile groups of participants without hypertension. Total PUFAs was more strongly associated with all-cause mortality in the second quartile for hypertension (HR: 0.70, 95% CI: 0.52, 0.95). The association of total PUFAs with mortality from IHD was stronger in participants aged ≥ 60 y (*P*-interaction = 0.013) and was protective in the second, third, and fourth quartile groups. There was no interaction between total PUFAs and subgroups in CVD mortality. The association of ω-3 with all-cause mortality was stronger in male (*P*-interaction = 0.031) and was protective in both the third and fourth quartile groups, with no interaction between CVD and IHD mortality and subgroups. There was no interaction between DHA and all-cause, CVD, and IHD mortality with each subgroup. ω-6 had different effects on risk of all-cause and IHD mortality in participants with and without hypertension (*P*-interaction = 0.005), although there was no statistical difference between them, they had a protective effect on risk of IHD mortality in participants aged ≥ 60 y (*P*-interaction = 0.004). Similar to ω-6, LA was protective against risk of mortality from IHD in participants ≥ 60 y of age (*P*-interaction = 0.008). There was no interaction between DHA and ω-6/ ω-3 ratio and all-cause mortality, CVD, and IHD mortality.

Among cluster 2 participants, the association of ω-3 and DHA with all-cause mortality was stronger in males, using the first quartile as the reference group (ω-3: *P*-interaction = 0.005; DHA: *P*-interaction = 0.013), and was a protective factor in the second, third, and fourth quartile groups. The effect of ω-6/ω-3 ratio on risk of all-cause mortality differed between males and females (*P*-interaction = 0.018), with risk of all-cause mortality for males in the highest quartile of ω-6/ω-3 ratio (HR: 1.65; 95% CI: 1.18, 2.31) was higher than that in female (HR: 1.36; 95% CI: 1.07-1.74). There was no interaction between exposure factors and risk of CVD mortality and between risk of IHD mortality and subgroups.

Among cluster 3 participants, using the lowest quartile as the reference group, only ω-6 and LA were found to have different effects on risk of IHD mortality in participants with or without hypertension (ω-6: *P*-interaction = 0.036; LA: *P*-interaction = 0.031). Decreased risk of mortality from IHD in participants without hypertension and an increased risk of mortality from IHD in participants with hypertension, although neither difference was statistically significant.

## Discussion

In this prospective study of 35,096 participants, we found an association between circulating PUFAs (total PUFAs, ω-3, DHA, ω-6, and LA) and risk of all-cause, CVD, and IHD mortality in patients with various CVD subtypes after categorization according to patient baseline characteristics.

This study found a significant nonlinear relationship between total PUFAs, ω-3, and DHA and risk for all-cause, CVD, and IHD mortality. In addition, higher levels of total PUFAs, ω-3, and DHA were associated with a lower risk of all-cause mortality. This is consistent with previous epidemiological investigations highlighting the role of total PUFAs and ω-3 in mortality risk [19]. A prospective cohort study found that replacing 5% of the energy in SFAs with PUFAs was associated with a 39% reduction in risk of all-cause mortality [20]. A recent meta-analysis involving 61,616 participants reconfirmed the association between circulating DHA levels and reduced risk of all-cause mortality [21]. In addition, studies in diabetic populations consistently reported that higher DHA intake was associated with a reduced risk of mortality from CVD [22]. And a large prospective study showed a 15% and 18% reduction in CVD mortality in males and females who consumed >0.18 g/d compared with those who consumed ω-3 <0.04 g/d, which complements our findings [23]. One possible mechanistic explanation for these associations is that ω-3 enhances fatty acid oxidation and inhibits acyl-CoA:1,2-diacylglycerol acyltransferase, thereby reducing lipogenesis and subsequently very low–density lipoprotein production in the liver, resulting in reduced triglyceride levels [24]. Cluster 2 has higher HDL-cholesterol, lower TG and ApoB:ApoA1 than cluster 1 and 3, and the mechanism of ω-3 intake has less effect on it.

However, it is important to acknowledge that not all studies have reported a positive effect of ω-3 on mortality risk [25]. Several studies, including randomized controlled trial and meta-analyses, have shown no significant protection against all-cause, CVD, and IHD mortality [[25], [26], [27]]. Our findings suggest that the mortality rates of ω-3 and DHA with CVD and IHD begin to trend upward at high intakes (fourth quartile array). The possible reason for this is that excess ω-3 and DHA may be predisposing factors for atrial fibrillation [28]. Another important result indicates a significant linear relationship between ω-6 and LA and risk of outcome. In cluster 3, ω-6 and LA were negatively associated with all-cause mortality but positively associated with CVD and IHD mortality, although these associations did not reach statistical significance. Current evidence on the effect of ω-6 on CVD risk is controversial. Although some studies have shown a favorable effect of ω-6 supplementation on CVD outcomes, conflicts remain [6]. Other studies have reported inconclusive results indicating no significant association between ω-6 intake and CVD risk [29], consistent with this result.

We also observed an inverse association between the highest quartile of LA levels and risk of all-cause mortality compared with the lowest quartile in cluster 2 only. Importantly, no such association was observed for CVD and IHD mortality. These findings are consistent with previous studies by Zhang et al. [30]. This may be due to the low number of deaths from CVD. It must be recognized that the relationship between ω-6 and CVD risk is multifaceted and may vary by individual characteristics and environment, adding complexity to our understanding of these associations.

Our study found a J-type exposure–response relationship between the ω-6/ω-3 ratio and risk of all-cause mortality, and only a significant linear relationship between CVD and IHD mortality. In cluster 1, an appropriate ω-6/ω-3 ratio (the second quartile array: 6.879–8.574) reduced risk of CVD mortality. In cluster 2, a higher ω-6/ω-3 ratio increased risk of all-cause mortality, independent of CVD and IHD mortality, and in cluster 3, a higher ω-6/ω-3 ratio was associated with an increased risk of all-cause, CVD, and IHD mortality. A higher ω-6/ω-3 ratio can increase risk of mortality, consistent with the findings of Zhang et al. [31], which divided the serum ω-6/ω-3 ratio in the population into quintiles and found that those in the highest quintile of serum ω-6/ω-3 ratios had 42% higher all-cause mortality and 40% higher CVD mortality compared with the first quartile, whereas the study by Zhang et al. showed that the adverse effect of a higher ω-6/ω-3 ratio on risk of mortality was only reflected in all-cause mortality, unrelated to CVD mortality [32]. In the cluster 3 population, the HbA1c value was higher, and the HbA1c value reflects the average blood sugar level over the past 2–3 mo and is an important indicator of diabetes management. Although there is less direct research, poor blood sugar control may affect overall inflammation levels and oxidative stress status. High ω-6/ω-3 ratios further promote inflammation and risk of mortality [33]. These results together suggest that the effect of the ω-6/ω-3 ratio at the same exposure dose may be significantly different for different CVD subtypes, and cluster 3 appears to be more susceptible to adverse effects of this ratio. These findings suggest that maintaining a reasonable ω-6/ω-3 ratio for overall health promotion outcomes.

### Strengths and limitations

This study offers several notable strengths. First, drawing from population cohorts, its prospective design is a significant advantage. It allows us to explore long-term associations and trends, enhancing the robustness of our findings. Second, using unsupervised ML techniques has enabled us to create fine-grained clusters of cardiovascular patients based on fundamental features while working with a relatively large and diverse sample size. Moreover, we have meticulously adjusted for various covariates, including demographics, lifestyle factors, chronic disease history, and medication history. This thorough adjustment process helps ensure that our findings are as robust as possible.

Despite its strengths, this study also presents several limitations that warrant consideration. First, PUFA biomarker concentrations were measured only once at baseline, and any changes in fatty acid levels over time were not incorporated into our analysis. This limitation could overlook dynamic variations in PUFAs status and their potential impact on the outcomes. Second, although our analysis accounted for numerous major risk factors, such as sociodemographic, lifestyle, clinical, and other dietary variables, it is vital to acknowledge the potential influence of residual confounders. These could stem from imprecise measurements or unknown factors not included in our models and may have affected the observed associations. For example, participants with higher PUFA status may have exhibited healthier dietary patterns or possessed higher socioeconomic status, which might have confounded the genuine associations. Finally, it is important to note that the majority of United Kingdom Biobank participants were of White British ethnicity. Therefore, the generalizability of our findings to more diverse or heterogeneous populations might be limited, and caution should be exercised when extrapolating these results to broader demographic groups.

In conclusion, in our comprehensive prospective analysis of the CVD population in the United Kingdom Biobank database, 3-cluster classifications of the CVD population were identified using available data. The effects of PUFAs on mortality outcomes were inconsistent in each cluster. These subtypes help to understand the heterogeneity of CVD and the role of PUFAs in mortality outcomes of different subtypes to inform population-specific investigative interventions based on clinical variables (such as lipids, blood sugar, inflammation, body composition), ultimately leading to better survival and prognostic outcomes. Future research is needed to validate the cluster classification in different data sets and confirm it, as well as he applicability of these clusters in clinical settings, and the intervention strategies of PUFAs for them.

## Author contributions

The authors’ responsibilities were as follows – J. Li, H. Li, H. Zheng, X. Chen, S. Ma, Q. Li, Z. Chen, H. Liang: designed research; J. Li, H. Zheng, X. Chen, J. Sun: analyzed data; J. Li, H. Li: wrote the article; J. Li, H. Li, Y. Li, D. Li and M. Lin: provided guidance on the subsequent revision. H. Liang, H. Li: had primary responsibility for the final content, and all authors: read and approved the final manuscript.

## Conflict of interest

The authors report no conflicts of interest.

## Funding

Supported by grants from National Science Foundation for Excellent Young Scholars (82122036), National Science Foundation for Young Scientists of China（82204396 and 82200558), Guangdong Provicial Medical Science and Technology Research Foundation (A2023006, A2023027). No funder influenced the study design, analyses, or interpretation of results. The views expressed in the article are those of the authors and not necessarily of the listed funders.

## Data availability

All the data are available on application to the UK Biobank to any researcher worldwide (www.ukbiobank.ac.uk).
