# Supplementary material for: Novel Classification of Cardiovascular Disease Subtypes Reveals Associations Between Mortality and Polyunsaturated Fatty Acids: Insights from the United Kingdom Biobank Study
Source: Curr Dev Nutr. 2024 Aug 5;8(9):104434. doi: 10.1016/j.cdnut.2024.104434 (PMC11403268; doi:10.1016/j.cdnut.2024.104434)
Supplement: Multimedia component 1 [file mmc1.docx]

**Novel Classification of Cardiovascular Disease Subtypes Reveals Associations Between Mortality and Polyunsaturated Fatty Acids: Insights from the UK Biobank Study**

Jiamei Li

**Supplementary Tables**

**Supplementary Table 1.** The numbers (percentages) of participants with missing variables.

**Supplementary Table 2.** Post-hoc comparison p-value of survival analysis by Log-rank test of Kaplan-Meier in participants.

**Supplementary Table 3.** The linear trend of the limiting cubic spline model of the relationship between total PUFAs, omega-3, DHA, omega-6, LA and omega-6/omega-3 ratio and all-cause, cardiovascular disease, and IHD mortality.

**Supplementary Table 4**. Subgroup analysis between total PUFAs, omega-3 PUFAs, DHA, omega-6 PUFAs, LA, and omega-6/omega-3 ratio and all-cause, cardiovascular disease, and IHD mortality in Cluster 1 (HR, 95%CI).

**Supplementary Table 5**. Subgroup analysis between total PUFAs, omega-3, DHA, omega-6, LA, and the omega-6/omega-3 ratio and all-cause, cardiovascular disease, and IHD mortality in Cluster 2 (HR, 95%CI).

**Supplementary Table 6**. Subgroup analysis between total PUFAs, omega-3, DHA, omega-6, LA, and omega-6/omega-3 ratio and all-cause, cardiovascular disease, and IHD mortality in Cluster 3 (HR, 95%CI).

**Supplementary Figure**

**Supplementary Figure 1.** Linear correlation between all pairs of features (Pearson correlation). BMI: body mass index; WHR: waist-hip ratio; DBP: diastolic blood pressure; SBP: systolic blood pressure; TC: total cholesterol; TG: total triglycerides; HDL-C: high-density lipoprotein-cholesterol; ApoB: ApoA1: apolipoprotein B: apolipoproteinA1; HbA1c: glycated hemoglobin; WBC: white blood cell count; PLT: platelet count.

**Supplementary Figure 2.** The box diagram of clustering indicators. BMI: body mass index; WHR: waist-hip ratio; DBP: diastolic blood pressure; SBP: systolic blood pressure; TC: total cholesterol; TG: total triglycerides; HDL-C: high-density lipoprotein-cholesterol; ApoB: ApoA1: apolipoprotein B: apolipoproteinA1; HbA1c: glycated hemoglobin; WBC: white blood cell count; PLT: platelet count.

**Supplementary Figure 3.** The distribution patterns of clustering variables were evaluated by creating Q-Q plots. BMI: body mass index; WHR: waist-hip ratio; DBP: diastolic blood pressure; SBP: systolic blood pressure; TC: total cholesterol; TG: total triglycerides; HDL-C: high-density lipoprotein-cholesterol; ApoB: ApoA1: apolipoprotein B: apolipoproteinA1; HbA1c: glycated hemoglobin; WBC: white blood cell count; PLT: platelet count.

**Supplementary Figure 4.** The distribution patterns of continuous variables were evaluated by creating Q-Q plots.

**Supplementary Table 1.** The numbers (percentages) of participants with missing variables.

| **Variables** | **n** | **%** |
| --- | --- | --- |
| Heart rate | 29 963 | 85.4 |
| Lp(a) | 11 005 | 31.4 |
| Physical activity | 7410 | 21.1 |
| Household income | 5741 | 16.4 |
| Albumin | 4 625 | 13.2 |
| C-reactive protein | 4 609 | 13.1 |
| HbA1c | 4313 | 12.3 |
| Creatinine | 2 146 | 6.11 |
| PLT | 1 642 | 4.68 |
| WBC | 1 615 | 4.60 |
| RBC | 1 342 | 3.82 |
| WHR | 1 281 | 3.65 |
| Hemoglobin concentration | 1 220 | 3.48 |
| BMI | 1 146 | 3.27 |
| TG | 778 | 2.22 |
| HDL-C | 727 | 2.07 |
| SBP | 549 | 1.56 |
| DBP | 481 | 1.37 |
| Waist circumference | 443 | 1.26 |
| ApoB: ApoA1 | 418 | 1.26 |
| LDL-C | 336 | 0.957 |
| TC | 245 | 0.698 |
| Smoking status | 213 | 0.607 |
| Alcohol status | 102 | 0.291 |
| Townsend deprivation index | 44 | 0.125 |

Abbreviations: Lp(a), lipoprotein (a); HbA1c, glycated hemoglobin; PLT, platelet count; WBC, white blood cell count; RBC, red blood cell count; WHR, waist-hip ratio; BMI, body mass index; TG, total triglycerides; HDL-C, high density lipoprotein-cholesterol; SBP, systolic blood pressure; DBP, diastolic blood pressure; ApoB: ApoA1, apolipoprotein B: apolipoprotein A1; LDL-C, low density lipoprotein-cholesterol; TC, total cholesterol; CVD, cardiovascular disease; IHD, ischemic heart disease.

**Supplementary Table 2.** Post-hoc comparison p-value of survival analysis by Log-rank test of Kaplan-Meier in participants.

| Comparison between clusters | All-cause mortality | CVD mortality | IHD mortality |
| --- | --- | --- | --- |
| Cluster 1 vs Cluster 2 | 0.49 | **0.039** | **<0.001** |
| Cluster 1 vs Cluster 3 | **<2e-16** | **<2e-16** | **<2e-16** |
| Cluster 2 vs Cluster 3 | **<2e-16** | **<2e-16** | **<2e-16** |

Abbreviations: CVD, cardiovascular disease; IHD, ischemic heart disease.

**Supplementary Table 3.** The linear trend of the limiting cubic spline model of the relationship between Total PUFAs, omega-3, DHA, omega-6, LA and omega-6/omega-3 ratio and all-cause, CVD, and IHD mortality.

| PUFAs | All-cause mortality | |  | CVD mortality | |  | IHD mortality | |
| --- | --- | --- | --- | --- | --- | --- | --- | --- |
|  | *P for nonlinearity* | *P for linearity* |  | *P for nonlinearity* | *P for linearity* |  | *P for nonlinearity* | *P for linearity* |
| Total PUFAs | **0.0079** | **<0.001** |  | **0.0083** | **<0.001** |  | **0.0195** | **<0.001** |
| Omega-3 | **<0.001** | **<0.001** |  | **<0.001** | **<0.001** |  | **<0.001** | **<0.001** |
| DHA | **<0.001** | **<0.001** |  | **<0.001** | **<0.001** |  | **<0.001** | **<0.001** |
| Omega-6 | 0.0915 | **<0.001** |  | 0.0581 | **<0.001** |  | 0.0630 | **<0.001** |
| LA | 0.1562 | **<0.001** |  | 0.2161 | **<0.001** |  | 0.0698 | **<0.001** |
| Omega-6/omega-3 ratio | **<0.001** | **<0.001** |  | 0.1134 | **<0.001** |  | 0.0613 | **<0.001** |

Abbreviations: PUFAs, polyunsaturated fatty acids; DHA, docosahexaenoic acid ; LA, linoleic acid; CVD, cardiovascular disease; IHD, ischemic heart disease.

**Supplementary Table 4**. Subgroup analysis between total PUFAs, omega-3, DHA, omega-6, LA, and omega-6/omega-3 ratio and all-cause, cardiovascular disease, and ischemic heart disease mortality in Cluster 1 (HR, 95%*CI*).

| **Subgroups** | **All-cause mortality** | | | | |  | **CVD mortality** | | | | |  | **IHD mortality** | | | | |
| --- | --- | --- | --- | --- | --- | --- | --- | --- | --- | --- | --- | --- | --- | --- | --- | --- | --- |
|  | **Q1** | **Q2** | **Q3** | **Q4** | ***P-int*** |  | **Q1** | **Q2** | **Q3** | **Q4** | ***P-int*** |  | **Q1** | **Q2** | **Q3** | **Q4** | ***P-int*** |
| **Total PUFAs** |  |  |  |  |  |  |  |  |  |  |  |  |  |  |  |  |  |
| Age (years) |  |  |  |  | 0.148 |  |  |  |  |  | 0.072 |  |  |  |  |  | **0.013** |
| <60 | Ref | 0.92  (0.60, 1.43) | 0.96  (0.63, 1.46) | 1.09  (0.72, 1.64) |  |  | Ref | 0.74  (0.33, 1.63) | 0.98  (0.47, 2.05) | 1.13  (0.56, 2.28) |  |  | Ref | 1.40  (0.45, 4.39) | 1.85  (0.62, 5.53) | 2.53  (0.88, 7.26) |  |
| ≥60 | Ref | 0.59  (0.41, 0.85)^**^ | 0.69  (0.49, 0.97)^*^ | 0.63  (0.45, 0.88)^**^ |  |  | Ref | 0.38  (0.19, 0.75)^**^ | 0.44  (0.24, 0.82)^*^ | 0.48  (0.26, 0.88)^*^ |  |  | Ref | 0.41  (0.18, 0.92)^*^ | 0.38  (0.18, 0.82)^*^ | 0.40  (0.19, 0.84)^*^ |  |
| Sex |  |  |  |  | 0.501 |  |  |  |  |  | 0.143 |  |  |  |  |  | 0.114 |
| Male | Ref | 0.69  (0.51, 0.94)^*^ | 0.76  (0.57, 1.01) | 0.69  (0.51, 0.94)^*^ |  |  | Ref | 0.55  (0.32, 0.95)^*^ | 0.66  (0.40, 1.10) | 0.67  (0.41, 1.10) |  |  | Ref | 0.65  (0.34, 1.25) | 0.63  (0.34, 1.19) | 0.76  (0.41, 1.39) |  |
| Female | Ref | 0.58  (0.26, 1.30) | 0.74  (0.35, 1.56) | 0.73  (0.35, 1.51) |  |  | Ref | 0.13  (0.02, 0.82)^*^ | 0.37  (0.10, 1.37) | 0.53  (0.16, 1.81) |  |  | Ref | 0.93  (0.00, Inf) | >99  (>99, >99) ^***^ | >99  (>99, >99) ^***^ |  |
| Diabetes |  |  |  |  | 0.548 |  |  |  |  |  | 0.350 |  |  |  |  |  | 0.653 |
| No | Ref | 0.67  (0.50, 0.91)^*^ | 0.71  (0.54, 0.94)^*^ | 0.73  (0.55, 0.96)^**^ |  |  | Ref | 0.52  (0.29, 0.95)^*^ | 0.69  (0.40, 1.19) | 0.78  (0.46, 1.32) |  |  | Ref | 0.62  (0.30, 1.28) | 0.71  (0.36, 1.39) | 0.86  (0.44, 1.66) |  |
| Yes | Ref | 0.67  (0.29, 1.56) | 1.05  (0.49, 2.27) | 0.74  (0.35, 1.58) |  |  | Ref | 0.50  (0.17, 1.50) | 0.31  (0.10, 0.94)^*^ | 0.41  (0.15, 1.09) |  |  | Ref | 1.03  (0.22, 4.72) | 0.62  (0.13, 3.02) | 0.87  (0.21, 3.57) |  |
| Hypertension |  |  |  |  | **0.014** |  |  |  |  |  | 0.146 |  |  |  |  |  | 0.079 |
| No | Ref | 0.48  (0.22, 1.04) | 0.37  (0.17, 0.80)^*^ | 0.34  (0.16, 0.72)^**^ |  |  | Ref | 1.47  (0.11, 19.27) | 2.62  (0.21, 33.32) | 1.73  (0.13, 22.63) |  |  | Ref | 3.44  (0.09, 125.14) | 2.41  (0.06, 96.78) | 2.68  (0.07, 106.05) |  |
| Yes | Ref | 0.70  (0.52, 0.95)^*^ | 0.83  (0.62, 1.10) | 0.81  (0.62, 1.08) |  |  | Ref | 0.49  (0.29, 0.84)^**^ | 0.61  (0.37, 1.00) | 0.70  (0.44, 1.12) |  |  | Ref | 0.65  (0.33, 1.29) | 0.76  (0.40, 1.44) | 0.92  (0.49, 1.70) |  |
| **Omega-3** |  |  |  |  |  |  |  |  |  |  |  |  |  |  |  |  |  |
| Age (years) |  |  |  |  | 0.178 |  |  |  |  |  | 0.179 |  |  |  |  |  | 0.983 |
| <60 | Ref | 0.82  (0.60, 1.11) | 0.76  (0.55, 1.03) | 0.83  (0.61, 1.13) |  |  | Ref | 0.82  (0.46, 1.46) | 0.85  (0.48, 1.51) | 0.99  (0.57, 1.71) |  |  | Ref | 0.89  (0.46, 1.74) | 1.01  (0.53, 1.94) | 0.76  (0.37, 1.52) |  |
| ≥60 | Ref | 1.09  (0.84, 1.42) | 0.71  (0.54, 0.93)^*^ | 0.85  (0.66, 1.09) |  |  | Ref | 0.83  (0.49, 1.42) | 0.47  (0.26, 0.84) | 0.86  (0.53, 1.40) |  |  | Ref | 1.04  (0.51, 2.11) | 0.54  (0.24, 1.20) | 1.16  (0.60, 2.23) |  |
| Sex |  |  |  |  | **0.031** |  |  |  |  |  | 0.067 |  |  |  |  |  | 0.158 |
| Male | Ref | 0.92  (0.74, 1.15) | 0.73  (0.58, 0.92)^**^ | 0.75  (0.60, 0.95)^*^ |  |  | Ref | 0.79  (0.52, 1.20) | 0.57  (0.37, 0.90)^*^ | 0.80  (0.53, 1.20) |  |  | Ref | 0.92  (0.55, 1.53) | 0.67  (0.39, 1.15) | 0.89  (0.54, 1.47) |  |
| Female | Ref | 1.22  (0.78, 1.90) | 0.74  (0.47, 1.18) | 1.05  (0.70, 1.59) |  |  | Ref | 1.46  (0.48, 4.45) | 1.27  (0.42, 3.83) | 2.22  (0.81, 6.06) |  |  | Ref | 1.76  (0.59, 5.23) | 2.15  (0.84, 5.55) | 2.53  (1.07, 6.02)^*^ |  |
| Diabetes |  |  |  |  | 0.484 |  |  |  |  |  | 0.837 |  |  |  |  |  | 0.711 |
| No | Ref | 1.00  (0.81, 1.24) | 0.72  (0.58, 0.90) | 0.84  (0.68, 1.03) |  |  | Ref | 0.82  (0.54, 1.27) | 0.65  (0.42, 1.01) | 0.97  (0.65, 1.44) |  |  | Ref | 0.88  (0.51, 1.49) | 0.76  (0.44, 1.30) | 1.01  (0.61, 1.68) |  |
| Yes | Ref. | 0.64  (0.35, 1.16) | 0.51  (0.28, 0.93) | 0.54  (0.30, 0.96) |  |  | Ref | 0.86  (0.34, 2.21) | 0.42  (0.15, 1.23) | 0.56  (0.21, 1.48) |  |  | Ref | 1.31  (0.41, 4.21) | 0.54  (0.14, 2.09) | 0.50  (0.13, 1.95) |  |
| Hypertension |  |  |  |  | 0.741 |  |  |  |  |  | 0.941 |  |  |  |  |  | 0.824 |
| No | Ref | 0.34  (0.18, 0.65)^**^ | 0.55  (0.32, 0.95)^*^ | 0.60  (0.35, 1.02) |  |  | Ref | 0.70  (0.14, 3.44) | 1.47  (0.37, 5.87) | 1.14  (0.27, 4.80) |  |  | Ref | 3.55  (0.12, 101.38) | 9.99  (0.30, 337.70) | 12.06  (0.38, 379.23) |  |
| Yes | Ref | 1.09  (0.89, 1.35) | 0.74  (0.59, 0.93)^**^ | 0.86  (0.70, 1.06) |  |  | Ref | 0.85  (0.57, 1.27) | 0.59  (0.38, 0.91)^*^ | 0.92  (0.63, 1.34) |  |  | Ref | 0.93  (0.57, 1.52) | 0.70  (0.41, 1.17) | 0.90  (0.55, 1.46) |  |
| **DHA** |  |  |  |  |  |  |  |  |  |  |  |  |  |  |  |  |  |
| Age (years) |  |  |  |  | 0.322 |  |  |  |  |  | 0.364 |  |  |  |  |  | 0.520 |
| <60 | Ref | 0.62  (0.46, 0.85)^**^ | 0.93  (0.70, 1.23) | 0.76  (0.55, 1.05) |  |  | Ref | 0.43  (0.24, 0.77)^**^ | 0.70  (0.41, 1.17) | 0.79  (0.46, 1.37) |  |  | Ref | 0.36  (0.18, 0.74)^**^ | 0.62  (0.34, 1.14) | 0.49  (0.24, 1.02) |  |
| ≥60 | Ref | 0.72  (0.28, 1.88) | 1.17  (0.50, 2.74) | 1.23  (0.54, 2.79) |  |  | Ref | 0.59  (0.35, 1.00)^*^ | 0.69  (0.42, 1.14) | 0.76  (0.47, 1.23) |  |  | Ref | 0.66  (0.33, 1.31) | 0.83  (0.43, 1.60) | 0.89  (0.47, 1.67) |  |
| Sex |  |  |  |  | 0.244 |  |  |  |  |  | 0.437 |  |  |  |  |  | 0.418 |
| Male | Ref | 0.77  (0.62,0.95)^*^ | 0.79  (0.63, 0.98)^*^ | 0.75  (0.60, 0.94)^*^ |  |  | Ref | 0.48  (0.31, 0.74)^**^ | 0.64  (0.42, 0.95)^*^ | 0.69  (0.46, 1.04) |  |  | Ref | 0.43  (0.25, 0.74)^**^ | 0.68  (0.43, 1.09) | 0.67  (0.41, 1.09) |  |
| Female | Ref | 1.12  (0.75, 1.68) | 1.07  (0.72, 1.59) | 0.97  (0.66, 1.43) |  |  | Ref | 0.72  (0.28, 1.88) | 1.17  (0.50, 2.74) | 1.23  (0.54, 2.79) |  |  | Ref | 0.77  (0.28, 2.10) | 0.77  (0.28, 2.11) | 0.62  (0.23, 1.68) |  |
| Diabetes |  |  |  |  | 0.348 |  |  |  |  |  | 0.979 |  |  |  |  |  | 0.661 |
| No | Ref | 0.83  (0.68, 1.01) | 0.84  (0.69, 1.03) | 0.79  (0.65, 0.97)^*^ |  |  | Ref | 0.54  (0.35, 0.83)^**^ | 0.78  (0.53, 1.15) | 0.81  (0.55, 1.20) |  |  | Ref | 0.51  (0.29, 0.87)^*^ | 0.83  (0.52, 1.33) | 0.74  (0.45, 1.21) |  |
| Yes | Ref | 0.74  (0.43, 1.26) | 0.70  (0.40, 1.24) | 0.65  (0.36, 1.17) |  |  | Ref | 0.35  (0.13, 0.91)^*^ | 0.28  (0.09, 0.85)^*^ | 0.46  (0.17, 1.23) |  |  | Ref | 0.32  (0.10, 1.01) | 0.10  (0.01, 0.78)^*^ | 0.23  (0.05, 1.08) |  |
| Hypertension |  |  |  |  | 0.707 |  |  |  |  |  | 0.954 |  |  |  |  |  | 0.532 |
| No | Ref | 0.92  (0.53, 1.60) | 0.79  (0.45, 1.40) | 0.97  (0.56, 1.68) |  |  | Ref | 1.79  (0.39, 8.14) | 2.62  (0.60, 11.47) | 1.39  (0.27, 7.12) |  |  | Ref | 5.37  (0.16, 179.75) | 12.20  (0.40, 372.87) | 13.59  (0.38, 490.78) |  |
| Yes | Ref | 0.81  (0.67, 0.99)^*^ | 0.83  (0.68, 1.01)^*^ | 0.76  (0.62, 0.94)^**^ |  |  | Ref | 0.46  (0.31, 0.70)^***^ | 0.63  (0.43, 0.92)^*^ | 0.75  (0.52, 1.09) |  |  | Ref | 0.45  (0.27, 0.74)^**^ | 0.62  (0.39, 0.98)^*^ | 0.60  (0.37, 0.97)^*^ |  |
| **Omega-6** |  |  |  |  |  |  |  |  |  |  |  |  |  |  |  |  |  |
| Age (years) |  |  |  |  | 0.265 |  |  |  |  |  | 0.111 |  |  |  |  |  | **0.004** |
| <60 | Ref | 0.88  (0.56, 1.41) | 0.85  (0.54, 1.33) | 1.10  (0.72, 1.70) |  |  | Ref | 1.13  (0.48, 2.67) | 1.07  (0.46, 2.50) | 1.36  (0.61, 3.06) |  |  | Ref | 2.90  (0.66, 12.75) | 2.56  (0.58, 11.28) | 4.14  (0.98, 17.44) |  |
| ≥60 | Ref | 0.87  (0.58, 1.30) | 0.86  (0.58, 1.25) | 0.85  (0.58, 1.23) |  |  | Ref | 0.36  (0.18, 0.72)^**^ | 0.44  (0.24, 0.82)^*^ | 0.47  (0.25, 0.86)^*^ |  |  | Ref | 0.35  (0.16, 0.77)^**^ | 0.38  (0.19, 0.79)^**^ | 0.30  (0.14, 0.63)^**^ |  |
| Sex |  |  |  |  | 0.955 |  |  |  |  |  | 0.308 |  |  |  |  |  | 0.182 |
| Male | Ref | 0.88  (0.63, 1.21) | 0.85  (0.62, 1.16) | 0.92  (0.68, 1.26) |  |  | Ref | 0.72  (0.41, 1.28) | 0.77  (0.45, 1.33) | 0.76  (0.44, 1.32) |  |  | Ref | 0.79  (0.40, 1.55) | 0.71  (0.37, 1.38) | 0.77  (0.40, 1.47) |  |
| Female | Ref | 0.67  (0.29, 1.57) | 0.70  (0.32, 1.55) | 0.75  (0.34, 1.62) |  |  | Ref | 0.10  (0.02, 0.56)^**^ | 0.16  (0.04, 0.54)^**^ | 0.32  (0.11, 0.95)^*^ |  |  | Ref | 0.00  (0.00, Inf) | 0.38  (0.14, 1.04) | 0.55  (0.22, 1.43) |  |
| Diabetes |  |  |  |  | 0.678 |  |  |  |  |  | 0.264 |  |  |  |  |  | 0.644 |
| No | Ref | 0.84  (0.61, 1.17) | 0.77  (0.56, 1.05) | 0.87  (0.64, 1.18) |  |  | Ref | 0.66  (0.35, 1.23) | 0.72  (0.40, 1.29) | 0.84  (0.47, 1.50) |  |  | Ref | 0.70  (0.33, 1.45) | 0.68  (0.34, 1.37) | 0.79  (0.39, 1.57) |  |
| Yes | Ref | 0.65  (0.27, 1.59) | 1.35  (0.63, 2.86) | 0.95  (0.45, 1.99) |  |  | Ref. | 0.43  (0.14, 1.32) | 0.40  (0.14, 1.14) | 0.41  (0.15, 1.08) |  |  | Ref | 0.81  (0.17, 3.82) | 0.77  (0.18, 3.35) | 0.85  (0.22, 3.36) |  |
| Hypertension |  |  |  |  | **0.005** |  |  |  |  |  | 0.083 |  |  |  |  |  | **0.028** |
| No | Ref | 1.03  (0.38, 2.76) | 0.81  (0.30, 2.15) | 0.67  (0.25, 1.79) |  |  | Ref | 1.26  (0.13, 11.96) | 1.10  (0.11, 10.49) | 1.18  (0.12, 11.25) |  |  | Ref | 1.44  (0.11, 18.52) | 0.27  (0.02, 4.80) | 0.46  (0.03, 7.89) |  |
| Yes | Ref | 0.83  (0.61, 1.15) | 0.85  (0.62, 1.15) | 0.93  (0.69, 1.25) |  |  | Ref | 0.58  (0.33, 1.01) | 0.64  (0.38, 1.07) | 0.73  (0.44, 1.20) |  |  | Ref | 0.70  (0.35, 1.40) | 0.76  (0.40, 1.47) | 0.85  (0.45, 1.62) |  |
| **LA** |  |  |  |  |  |  |  |  |  |  |  |  |  |  |  |  |  |
| Age (years) |  |  |  |  | 0.353 |  |  |  |  |  | 0.250 |  |  |  |  |  | **0.008** |
| <60 | Ref | 0.91  (0.55, 1.50) | 0.86  (0.53, 1.39) | 1.09  (0.69, 1.74) |  |  | Ref | 1.31  (0.49, 3.51) | 1.27  (0.48, 3.34) | 1.64  (0.64, 4.19) |  |  | Ref | 4.03  (0.52, 30.91) | 3.85  (0.51, 29.16) | 6.93  (0.94, 50.93) |  |
| ≥60 | Ref | 0.92  (0.61, 1.39) | 0.86  (0.58, 1.28) | 0.83  (0.56, 1.22) |  |  | Ref | 0.47  (0.23, 0.95) | 0.41  (0.21, 0.81) | 0.57  (0.30, 1.09) |  |  | Ref | 0.52  (0.23, 1.18) | 0.36  (0.16, 0.80)^*^ | 0.39  (0.18, 0.86)^*^ |  |
| Sex |  |  |  |  | 0.683 |  |  |  |  |  | 0.498 |  |  |  |  |  | 0.210 |
| Male | Ref | 0.94  (0.67, 1.33) | 0.80  (0.57, 1.12) | 0.92  (0.66, 1.27) |  |  | Ref | 0.88  (0.47, 1.65) | 0.78  (0.42, 1.44) | 0.93  (0.51, 1.70) |  |  | Ref | 0.98  (0.46, 2.06) | 0.70  (0.33, 1.47) | 0.98  (0.46, 2.06) |  |
| Female | Ref | 0.60  (0.26, 1.35) | 0.83  (0.40, 1.74) | 0.74  (0.36, 1.53) |  |  | Ref | 0.11  (0.02, 0.64)^*^ | 0.18  (0.05, 0.64)^*^ | 0.40  (0.13, 1.20) |  |  | Ref | 0.00  (0.00, Inf) | 0.39  (0.14, 1.08) | 0.60  (0.23, 1.54) |  |
| Diabetes |  |  |  |  | 0.467 |  |  |  |  |  | 0.283 |  |  |  |  |  | 0.722 |
| No | Ref | 0.89  (0.63, 1.24) | 0.78  (0.56, 1.08) | 0.85  (0.61, 1.17) |  |  | Ref | 0.83  (0.42, 1.63) | 0.75  (0.39, 1.45) | 1.09  (0.58, 2.07) |  |  | Ref | 1.00  (0.43, 2.30) | 0.74  (0.32, 1.69) | 1.15  (0.52, 2.56) |  |
| Yes | Ref | 0.79  (0.31, 2.01) | 1.31  (0.57, 2.99) | 1.07  (0.47, 2.43) |  |  | Ref | 0.49  (0.16, 1.50) | 0.41  (0.14, 1.18) | 0.36  (0.13, 1.03) |  |  | Ref | 0.69  (0.15, 3.19) | 0.72  (0.17, 2.99) | 0.62  (0.15, 2.56) |  |
| Hypertension |  |  |  |  | **0.004** |  |  |  |  |  | 0.062 |  |  |  |  |  | **0.017** |
| No | Ref | 0.77  (0.30, 1.99) | 0.58  (0.23, 1.45) | 0.44  (0.17, 1.10) |  |  | Ref | >99   (0.00, >99) | >99  (0.00, >99) | >99  (0.00, >99  ) |  |  | Ref | >99  (0.00, >99) | >99  (0.00, >99) | >99  (0.00, >99) |  |
| Yes | Ref | 0.90  (0.64, 1.25) | 0.87  (0.63, 1.20) | 0.94  (0.69, 1.30) |  |  | Ref | 0.59  (0.33, 1.06) | 0.59  (0.34, 1.02) | 0.78  (0.46, 1.32) |  |  | Ref | 0.72  (0.35, 1.51) | 0.66  (0.32, 1.34) | 0.94  (0.47, 1.86) |  |
| **Omega-6/3** |  |  |  |  |  |  |  |  |  |  |  |  |  |  |  |  |  |
| Age (years) |  |  |  |  | 0.478 |  |  |  |  |  | 0.627 |  |  |  |  |  | 0.331 |
| <60 | Ref | 0.72  (0.50, 1.02) | 0.84  (0.61, 1.17) | 1.12  (0.82, 1.52) |  |  | Ref | 0.46  (0.23, 0.91) | 1.02  (0.59, 1.77) | 0.90  (0.51, 1.57) |  |  | Ref | 0.63  (0.26, 1.53) | 1.58  (0.77, 3.25) | 1.49  (0.72, 3.10) |  |
| ≥60 | Ref | 0.88  (0.69, 1.12) | 1.03  (0.82, 1.31) | 1.15  (0.91, 1.46) |  |  | Ref | 0.72  (0.43, 1.21) | 0.95  (0.59, 1.55) | 1.05  (0.64, 1.71) |  |  | Ref | 0.67  (0.35, 1.28) | 0.72  (0.38, 1.36) | 0.77  (0.40, 1.46) |  |
| Sex |  |  |  |  | 0.129 |  |  |  |  |  | 0.169 |  |  |  |  |  | 0.729 |
| Male | Ref | 0.84  (0.66, 1.06) | 0.99  (0.79, 1.24) | 1.25  (1.00, 1.56) |  |  | Ref | 0.58  (0.36, 0.95) | 0.96  (0.63, 1.47) | 1.11  (0.73, 1.67) |  |  | Ref | 0.66  (0.38, 1.16) | 0.99  (0.60, 1.64) | 1.09  (0.66, 1.80) |  |
| Female | Ref | 0.83  (0.59, 1.17) | 0.98  (0.70, 1.39) | 0.95  (0.66, 1.37) |  |  | Ref | 0.68  (0.31, 1.51) | 1.16  (0.56, 2.37) | 0.45  (0.18, 1.14) |  |  | Ref | 0.51  (0.15, 1.72) | 1.43  (0.59, 3.45) | 0.56  (0.19, 1.68) |  |
| Diabetes |  |  |  |  | 0.384 |  |  |  |  |  | 0.595 |  |  |  |  |  | 0.653 |
| No | Ref | 0.84  (0.68, 1.03) | 0.98  (0.80, 1.20) | 1.14  (0.93, 1.39) |  |  | Ref | 0.60  (0.39, 0.93)^*^ | 0.92  (0.62, 1.37) | 0.94  (0.63, 1.40) |  |  | Ref | 0.65  (0.38, 1.12) | 0.91  (0.55, 1.49) | 0.95  (0.57, 1.56) |  |
| Yes | Ref | 0.79  (0.42, 1.46) | 1.02  (0.57, 1.83) | 1.55  (0.90, 2.67) |  |  | Ref | 0.61  (0.18, 2.05) | 1.71  (0.67, 4.41) | 1.42  (0.53, 3.77) |  |  | Ref | 0.33  (0.04, 3.03) | 2.63  (0.76, 9.12) | 2.11  (0.58, 7.59) |  |
| Hypertension |  |  |  |  | 0.549 |  |  |  |  |  | 0.352 |  |  |  |  |  | 0.188 |
| No | Ref | 0.69  (0.38, 1.25) | 0.79  (0.45, 1.39) | 1.13  (0.67, 1.92) |  |  | Ref | 0.40  (0.08, 1.98) | 1.32  (0.42, 4.14) | 0.63  (0.16, 2.51) |  |  | Ref | 0.60  (0.10, 3.54) | 0.28  (0.04, 1.84) | 0.23  (0.02, 2.42) |  |
| Yes | Ref | 0.84  (0.68, 1.04) | 1.00  (0.82, 1.22) | 1.16  (0.95, 1.42) |  |  | Ref | 0.62  (0.40, 0.95)^*^ | 0.96  (0.66, 1.41) | 0.98  (0.67, 1.44) |  |  | Ref | 0.65  (0.37, 1.11) | 1.10  (0.68, 1.77) | 1.09  (0.67, 1.77) |  |

Adjusted for sex, age, Townsend deprivation index, household income, physical activity, smoking status, alcohol status, cholesterol-lowering medication use, antihypertensive drugs use, insulin treatment, aspirin use, diabetes, and hypertension. HR: hazard ratio; CI: confidence interval; PUFAs: polyunsaturated fatty acids; DHA, docosahexaenoic acid; LA, linoleic acid; CVD, cardiovascular disease; IHD, ischemic heart disease.

**Supplementary Table 5**. Subgroup analysis between total PUFAs, omega-3, DHA, omega-6, LA, and omega-6/omega-3 ratio and all-cause, cardiovascular disease, and ischemic heart disease mortality in Cluster 2 (HR, 95%CI).

| **Subgroups** | **All-cause mortality** | | | | |  | **CVD mortality** | | | | |  | **IHD mortality** | | | | |
| --- | --- | --- | --- | --- | --- | --- | --- | --- | --- | --- | --- | --- | --- | --- | --- | --- | --- |
|  | **Q1** | **Q2** | **Q3** | **Q4** | ***P-int*** |  | **Q1** | **Q2** | **Q3** | **Q4** | ***P-int*** |  | **Q1** | **Q2** | **Q3** | **Q4** | ***P-int*** |
| **Total PUFAs** |  |  |  |  |  |  |  |  |  |  |  |  |  |  |  |  |  |
| Age (years) |  |  |  |  | 0.568 |  |  |  |  |  | 0.365 |  |  |  |  |  | 0.954 |
| <60 | Ref | 0.83  (0.58, 1.20) | 0.90  (0.62, 1.30) | 0.92  (0.61, 1.39) |  |  | Ref | 1.54  (0.64, 3.70) | 0.65  (0.23, 1.88) | 0.50  (0.14, 1.76) |  |  | Ref | 2.78  (0.54, 14.30) | 0.84  (0.11, 6.33) | 1.28  (0.16, 10.26) |  |
| ≥60 | Ref | 0.83  (0.65, 1.04) | 0.87  (0.69, 1.10) | 0.80  (0.62, 1.03) |  |  | Ref | 0.75  (0.46, 1.24) | 0.94  (0.58, 1.54) | 0.93  (0.55, 1.58) |  |  | Ref | 0.75  (0.39, 1.45) | 0.75  (0.37, 1.50) | 1.19  (0.59, 2.41) |  |
| Sex |  |  |  |  | 0.095 |  |  |  |  |  | 0.408 |  |  |  |  |  | 0.636 |
| Male | Ref | 0.87  (0.66, 1.16) | 0.68  (0.50, 0.94)^*^ | 0.75  (0.51, 1.11) |  |  | Ref | 0.95  (0.52, 1.74) | 0.89  (0.46, 1.71) | 1.14  (0.54, 2.43) |  |  | Ref | 1.27  (0.58, 2.79) | 1.12  (0.48, 2.62) | 1.63  (0.64, 4.18) |  |
| Female | Ref | 0.75  (0.57, 0.99)^*^ | 0.91  (0.70, 1.19) | 0.78  (0.60, 1.03) |  |  | Ref | 0.84  (0.45, 1.56) | 0.85  (0.46, 1.57) | 0.71  (0.37, 1.35) |  |  | Ref | 0.48  (0.19, 1.23) | 0.37  (0.13, 1.03) | 0.72  (0.29, 1.78) |  |
| Diabetes |  |  |  |  | 0.945 |  |  |  |  |  | 0.981 |  |  |  |  |  | 0.263 |
| No | Ref | 0.80  (0.65, 0.99)^*^ | 0.85  (0.70, 1.05) | 0.79  (0.64, 0.99)^*^ |  |  | Ref | 0.94  (0.60, 1.48) | 0.91  (0.57, 1.46) | 0.87  (0.52, 1.45) |  |  | Ref | 1.13  (0.58, 2.18) | 0.85  (0.41, 1.77) | 1.49  (0.72, 3.08) |  |
| Yes | Ref | 0.75  (0.39, 1.47) | 0.58  (0.26, 1.30) | 0.45  (0.18, 1.13) |  |  | Ref | 0.55  (0.11, 2.66) | 0.80  (0.15, 4.24) | 0.52  (0.07, 3.61) |  |  | Ref | 0.09  (0.01, 1.54) | 0.38  (0.05, 3.22) | 0.11  (0.01, 1.80) |  |
| Hypertension |  |  |  |  | 0.692 |  |  |  |  |  | 0.685 |  |  |  |  |  | 0.171 |
| No | Ref | 0.50  (0.32, 0.79)^**^ | 0.73  (0.48, 1.11) | 0.66  (0.42, 1.06) |  |  | Ref | 0.50  (0.17, 1.44) | 0.50  (0.17, 1.48) | 0.78  (0.27, 2.27) |  |  | Ref | 1.12  (0.13, 9.30) | 1.06  (0.13, 8.66) | 4.12  (0.56, 30.26) |  |
| Yes | Ref | 0.89  (0.71, 1.11) | 0.86  (0.69, 1.08) | 0.79  (0.62, 1.01) |  |  | Ref | 0.99  (0.62, 1.60) | 0.98  (0.60, 1.60) | 0.84  (0.49, 1.45) |  |  | Ref | 0.89  (0.47, 1.67) | 0.70  (0.35, 1.40) | 0.95  (0.46, 1.95) |  |
| **Omega-3** |  |  |  |  |  |  |  |  |  |  |  |  |  |  |  |  |  |
| Age (years) |  |  |  |  | 0.704 |  |  |  |  |  | 0.340 |  |  |  |  |  | 0.487 |
| <60 | Ref | 0.78  (0.55, 1.10) | 0.62  (0.42, 0.93)^*^ | 0.84  (0.57, 1.24) |  |  | Ref | 0.67  (0.29, 1.57) | 0.54  (0.20, 1.44) | 0.42  (0.13, 1.32) |  |  | Ref | 0.86  (0.19, 3.94) | 0.80  (0.14, 4.64) | 0.88  (0.16, 5.01) |  |
| ≥60 | Ref | 0.78  (0.62, 0.98)^*^ | 0.76  (0.61, 0.96)^*^ | 0.74  (0.59, 0.92)^**^ |  |  | Ref | 0.71  (0.42, 1.19) | 0.87  (0.54, 1.42) | 0.98  (0.61, 1.56) |  |  | Ref | 1.12  (0.58, 2.16) | 0.91  (0.45, 1.85) | 0.93  (0.46, 1.88) |  |
| Sex |  |  |  |  | **0.005** |  |  |  |  |  | 0.748 |  |  |  |  |  | 0.811 |
| Male | Ref | 0.61  (0.46, 0.83)^**^ | 0.63  (0.46, 0.88)^**^ | 0.54  (0.38, 0.77)^**^ |  |  | Ref | 0.58  (0.30, 1.14) | 0.81  (0.42, 1.57) | 0.96  (0.51, 1.82) |  |  | Ref | 0.96  (0.45, 2.04) | 0.81  (0.34, 1.93) | 0.99  (0.43, 2.32) |  |
| Female | Ref | 0.87  (0.67, 1.13) | 0.77  (0.60, 1.00)^*^ | 0.79  (0.61, 1.01) |  |  | Ref | 0.76  (0.41, 1.39) | 0.75  (0.41, 1.34) | 0.75  (0.42, 1.33) |  |  | Ref | 1.28  (0.44, 3.76) | 0.93  (0.32, 2.74) | 0.87  (0.30, 2.57) |  |
| Diabetes |  |  |  |  | 0.543 |  |  |  |  |  | 0.557 |  |  |  |  |  | 0.878 |
| No | Ref | 0.73  (0.60, 0.89)^**^ | 0.70  (0.57, 0.85)^***^ | 0.69  (0.57, 0.84)^***^ |  |  | Ref | 0.72  (0.46, 1.14) | 0.81  (0.52, 1.27) | 0.81  (0.52, 1.26) |  |  | Ref | 1.38  (0.72, 2.61) | 1.02  (0.50, 2.06) | 0.97  (0.48, 1.97) |  |
| Yes | Ref | 0.75  (0.36, 1.56) | 0.63  (0.28, 1.42) | 0.68  (0.30, 1.53) |  |  | Ref | 0.31  (0.05, 2.01) | 0.43  (0.07, 2.61) | 0.77  (0.16, 3.76) |  |  | Ref | 0.00 (0.00, >99) | 0.20 (0.02, 1.87) | 0.18  (0.02, 1.43) |  |
| Hypertension |  |  |  |  | 0.269 |  |  |  |  |  | 0.381 |  |  |  |  |  | 0.287 |
| No | Ref | 0.92  (0.61, 1.41) | 0.88  (0.57, 1.37) | 0.75  (0.47, 1.18) |  |  | Ref | 0.64  (0.21, 1.94) | 0.84  (0.28, 2.47) | 1.03  (0.37, 2.85) |  |  | Ref | 1.23  (0.16, 9.62) | 1.88  (0.27, 13.16) | 1.74  (0.25, 12.27) |  |
| Yes | Ref | 0.70  (0.56, 0.86)^**^ | 0.66  (0.53, 0.81)^***^ | 0.67  (0.54, 0.83)^***^ |  |  | Ref | 0.70  (0.43, 1.13) | 0.76  (0.47, 1.21) | 0.77  (0.48, 1.22) |  |  | Ref | 1.08 (0.58, 2.02) | 0.82  (0.41, 1.63) | 0.82  (0.41, 1.63) |  |
| **DHA** |  |  |  |  |  |  |  |  |  |  |  |  |  |  |  |  |  |
| Age (years) |  |  |  |  | 0.854 |  |  |  |  |  | 0.340 |  |  |  |  |  | 0.325 |
| <60 | Ref | 0.74  (0.52, 1.08) | 0.74  (0.50, 1.08) | 0.70  (0.47, 1.03) |  |  | Ref | 0.41  (0.16, 1.05) | 0.54  (0.22, 1.30) | 0.36  (0.13, 1.01) |  |  | Ref | 0.27  (0.05, 1.47) | 0.60  (0.14, 2.50) | 0.15  (0.02, 1.37) |  |
| ≥60 | Ref | 0.76  (0.58, 0.99)^*^ | 0.72  (0.55, 0.93)^**^ | 0.74  (0.58, 0.95)^*^ |  |  | Ref | 0.75  (0.41, 1.38) | 0.78  (0.45, 1.38) | 1.01  (0.59, 1.73) |  |  | Ref | 0.61  (0.27, 1.37) | 0.90  (0.44, 1.85) | 0.77  (0.37, 1.60) |  |
| Sex |  |  |  |  | **0.013** |  |  |  |  |  | 0.944 |  |  |  |  |  | 0.939 |
| Male | Ref | 0.61  (0.45, 0.84)^**^ | 0.64  (0.47, 0.88)^**^ | 0.50  (0.35, 0.69)^***^ |  |  | Ref | 0.62  (0.31, 1.26) | 0.68  (0.34, 1.34) | 0.78  (0.40, 1.51) |  |  | Ref | 0.36  (0.14, 0.90)^*^ | 0.70  (0.32, 1.52) | 0.52  (0.23, 1.21) |  |
| Female | Ref | 0.85  (0.63, 1.16) | 0.76  (0.56, 1.02) | 0.81  (0.61, 1.08) |  |  | Ref | 0.60  (0.29, 1.24) | 0.67  (0.35, 1.30) | 0.77  (0.41, 1.46) |  |  | Ref | 1.05  (0.27, 4.11) | 1.16  (0.33, 4.12) | 0.96  (0.27, 3.44) |  |
| Diabetes |  |  |  |  | 0.559 |  |  |  |  |  | 0.882 |  |  |  |  |  | 0.675 |
| No | Ref | 0.70  (0.56, 0.88)^**^ | 0.66  (0.53, 0.82)^***^ | 0.66  (0.54, 0.82)^***^ |  |  | Ref | 0.64  (0.38, 1.10) | 0.81  (0.49, 1.33) | 0.82  (0.51, 1.34) |  |  | Ref | 0.62  (0.28, 1.38) | 1.12  (0.55, 2.27) | 0.70  (0.33, 1.48) |  |
| Yes | Ref | 0.96  (0.40, 2.30) | 0.74  (0.33, 1.67) | 0.76  (0.33, 1.76) |  |  | Ref | 0.32  (0.06, 1.83) | 0.00 (0.00, >99) | 0.33  (0.07, 1.54) |  |  | Ref | 0.03  (0.00, 0.56)^*^ | 0.00 (0.00, >99) | 0.07  (0.01, 0.67)^*^ |  |
| Hypertension |  |  |  |  | 0.190 |  |  |  |  |  | 0.588 |  |  |  |  |  | 0.604 |
| No | Ref | 0.63  (0.38, 1.04) | 0.91  (0.57, 1.44) | 0.73  (0.46, 1.17) |  |  | Ref | 0.95  (0.25, 3.64) | 1.51  (0.45, 5.04) | 1.14  (0.33, 3.98) |  |  | Ref | 0.00 (0.00, >99) | 2.71  (0.31, 24.04) | 0.94  (0.08, 10.49) |  |
| Yes | Ref | 0.73  (0.57, 0.93)^*^ | 0.62  (0.49, 0.78)^***^ | 0.65  (0.51, 0.81)^***^ |  |  | Ref | 0.56  (0.32, 0.96)^*^ | 0.57  (0.34, 0.95)^*^ | 0.70  (0.43, 1.14) |  |  | Ref | 0.51  (0.24, 1.06) | 0.65  (0.33, 1.28) | 0.54  (0.27, 1.07) |  |
| **Omega-6** |  |  |  |  |  |  |  |  |  |  |  |  |  |  |  |  |  |
| Age (years) |  |  |  |  | 0.495 |  |  |  |  |  | 0.453 |  |  |  |  |  | 0.802 |
| <60 | Ref | 0.84  (0.57, 1.22) | 0.56  (0.32, 0.96) | 1.03  (0.69, 1.54) |  |  | Ref | 0.98  (0.39, 2.48) | 0.76  (0.28, 2.04) | 0.76  (0.25, 2.24) |  |  | Ref | 1.18  (0.19, 7.23) | 1.17  (0.18, 7.48) | 2.16  (0.34, 13.55) |  |
| ≥60 | Ref | 0.94  (0.74, 1.18) | 0.96  (0.76, 1.22) | 0.90  (0.70, 1.17) |  |  | Ref | 0.88  (0.53, 1.46) | 1.29  (0.79, 2.10) | 0.85  (0.48, 1.50) |  |  | Ref | 0.98  (0.51, 1.91) | 1.09  (0.54, 2.18) | 1.26  (0.59, 2.69) |  |
| Sex |  |  |  |  | 0.357 |  |  |  |  |  | 0.386 |  |  |  |  |  | 0.607 |
| Male | Ref | 0.91  (0.68, 1.22) | 0.79  (0.58, 1.09) | 0.82  (0.55, 1.21) |  |  | Ref | 1.16  (0.62, 2.15) | 1.18  (0.61, 2.28) | 1.08  (0.47, 2.47) |  |  | Ref | 1.45  (0.65, 3.23) | 1.45  (0.62, 3.42) | 1.33  (0.46, 3.82) |  |
| Female | Ref | 0.85  (0.64, 1.12) | 0.95  (0.73, 1.25) | 0.91  (0.69, 1.20) |  |  | Ref | 0.69  (0.36, 1.29) | 1.08  (0.60, 1.95) | 0.68  (0.36, 1.31) |  |  | Ref | 0.46  (0.17, 1.29) | 0.66  (0.25, 1.76) | 1.15  (0.45, 2.92) |  |
| Diabetes |  |  |  |  | 0.730 |  |  |  |  |  | 0.859 |  |  |  |  |  | 0.161 |
| No | Ref | 0.93  (0.75, 1.15) | 0.95  (0.77, 1.17) | 0.94  (0.75, 1.18) |  |  | Ref | 0.88  (0.55, 1.42) | 1.24  (0.78, 1.95) | 0.84  (0.49, 1.43) |  |  | Ref | 1.10  (0.54, 2.25) | 1.41  (0.69, 2.87) | 1.77  (0.83, 3.81) |  |
| Yes | Ref | 0.58  (0.31, 1.11) | 0.39  (0.16, 0.96)^*^ | 0.49  (0.21, 1.15) |  |  | Ref. | 1.06  (0.29, 3.82) | 0.36  (0.03, 3.81) | 0.65  (0.10, 4.32) |  |  | Ref | 0.67  (0.16, 2.86) | 0.00 (0.00, >99) | 0.19  (0.02, 2.35) |  |
| Hypertension |  |  |  |  | 0.972 |  |  |  |  |  | 0.866 |  |  |  |  |  | 0.199 |
| No | Ref | 0.73  (0.47, 1.13) | 0.72  (0.46, 1.11) | 0.78  (0.48, 1.24) |  |  | Ref | 0.95  (0.33, 2.73) | 1.00  (0.35, 2.82) | 0.58  (0.16, 2.15) |  |  | Ref | 1.73  (0.20, 14.77) | 1.92  (0.27, 13.51) | 3.80  (0.46, 31.31) |  |
| Yes | Ref | 0.93  (0.75, 1.17) | 0.94  (0.75, 1.19) | 0.90  (0.70, 1.15) |  |  | Ref | 0.88  (0.54, 1.44) | 1.20  (0.74, 1.94) | 0.85  (0.49, 1.48) |  |  | Ref | 0.97  (0.50, 1.86) | 1.00  (0.50, 2.01) | 1.17  (0.56, 2.46) |  |
| **LA** |  |  |  |  |  |  |  |  |  |  |  |  |  |  |  |  |  |
| Age (years) |  |  |  |  | 0.699 |  |  |  |  |  | 0.347 |  |  |  |  |  | 0.766 |
| <60 | Ref | 0.87  (0.60, 1.27) | 0.91  (0.62, 1.33) | 0.89  (0.58, 1.37) |  |  | Ref | 0.77  (0.31, 1.91) | 0.72  (0.28, 1.85) | 0.47  (0.15, 1.51) |  |  | Ref | 1.35  (0.24, 7.57) | 1.08  (0.17, 6.96) | 1.53  (0.22, 10.52) |  |
| ≥60 | Ref | 0.86  (0.69, 1.07) | 0.83  (0.66, 1.05) | 0.81  (0.63, 1.05) |  |  | Ref | 0.92  (0.57, 1.48) | 1.15  (0.71, 1.87) | 1.04  (0.60, 1.81) |  |  | Ref | 0.69  (0.35, 1.36) | 1.23  (0.65, 2.35) | 1.28  (0.61, 2.69) |  |
| Sex |  |  |  |  | 0.623 |  |  |  |  |  | 0.304 |  |  |  |  |  | 0.588 |
| Male | Ref | 0.80  (0.60, 1.07) | 0.76  (0.56, 1.04) | 0.72  (0.48, 1.07) |  |  | Ref | 0.99  (0.54, 1.81) | 1.08  (0.57, 2.05) | 0.86  (0.37, 2.02) |  |  | Ref | 0.98  (0.44, 2.19) | 1.55  (0.70, 3.41) | 0.96  (0.32, 2.87) |  |
| Female | Ref | 0.88  (0.68, 1.13) | 0.90  (0.70, 1.17) | 0.86  (0.65, 1.13) |  |  | Ref | 0.78  (0.43, 1.41) | 1.02 (0.57, 1.83) | 0.87  (0.46, 1.65) |  |  | Ref | 0.40  (0.15, 1.13) | 0.76  (0.30, 1.94) | 1.40  (0.55, 3.52) |  |
| Diabetes |  |  |  |  | 0.878 |  |  |  |  |  | 0.732 |  |  |  |  |  | 0.282 |
| No | Ref | 0.87  (0.72, 1.07) | 0.86  (0.70, 1.05) | 0.82  (0.65, 1.02) |  |  | Ref | 0.91  (0.58, 1.42) | 1.11  (0.71, 1.73) | 0.90  (0.54, 1.52) |  |  | Ref | 0.90  (0.45, 1.80) | 1.47  (0.76, 2.86) | 1.64  (0.77, 3.48) |  |
| Yes | Ref | 0.56  (0.28, 1.10) | 0.65  (0.30, 1.43) | 0.64  (0.26, 1.57) |  |  | Ref | 0.71  (0.17, 2.91) | 0.83  (0.12, 5.67) | 0.74  (0.11, 4.92) |  |  | Ref | 0.33  (0.05, 2.24) | 0.38  (0.03, 5.03) | 0.23  (0.02, 2.99) |  |
| Hypertension |  |  |  |  | 0.706 |  |  |  |  |  | 0.663 |  |  |  |  |  | 0.277 |
| No | Ref | 0.76  (0.49, 1.16) | 0.53  (0.18, 1.59) | 0.82  (0.51, 1.31) |  |  | Ref | 0.53  (0.18, 1.59) | 0.94  (0.35, 2.53) | 0.46  (0.13, 1.68) |  |  | Ref | 0.60  (0.05, 7.72) | 3.76  (0.49, 28.79) | 4.82  (0.48, 48.12) |  |
| Yes | Ref | 0.87  (0.70, 1.08) | 0.90  (0.72, 1.11) | 0.77  (0.60, 0.99)^*^ |  |  | Ref | 0.97  (0.61, 1.53) | 1.07  (0.67, 1.72) | 0.98  (0.57, 1.68) |  |  | Ref | 0.76  (0.40, 1.46) | 1.03  (0.54, 1.97) | 1.07  (0.51, 2.25) |  |
| **Omega-6/3** |  |  |  |  |  |  |  |  |  |  |  |  |  |  |  |  |  |
| Age (years) |  |  |  |  | 0.711 |  |  |  |  |  | 0.460 |  |  |  |  |  | 0.654 |
| <60 | Ref | 0.79  (0.50, 1.26) | 0.87  (0.56, 1.35) | 1.34  (0.91, 1.98) |  |  | Ref | 0.82  (0.24, 2.83) | 0.82  (0.24, 2.86) | 2.18  (0.78, 6.08) |  |  | Ref | 2.66  (0.27, 26.00) | 0.82  (0.05, 13.27) | 3.39  (0.38, 29.91) |  |
| ≥60 | Ref | 1.04  (0.84, 1.29) | 1.17  (0.95, 1.45) | 1.29  (1.04, 1.62)^*^ |  |  | Ref | 0.85  (0.54, 1.32) | 0.76  (0.48, 1.22) | 0.97  (0.61, 1.54) |  |  | Ref | 1.03  (0.53, 2.00) | 1.07  (0.55, 2.08) | 1.34  (0.69, 2.60) |  |
| Sex |  |  |  |  | **0.018** |  |  |  |  |  | 0.294 |  |  |  |  |  | 0.440 |
| Male | Ref | 1.02  (0.69, 1.51) | 1.21  (0.85, 1.74) | 1.65  (1.18, 2.31)^**^ |  |  | Ref | 0.68  (0.32, 1.44) | 0.47  (0.22, 1.02) | 1.20  (0.65, 2.20) |  |  | Ref | 0.82  (0.31, 2.19) | 0.71  (0.28, 1.82) | 1.47  (0.66, 3.28) |  |
| Female | Ref | 1.04  (0.83, 1.30) | 1.16  (0.92, 1.46) | 1.36  (1.07, 1.74)^*^ |  |  | Ref | 0.96  (0.58, 1.61) | 1.02  (0.60, 1.73) | 1.13  (0.63, 2.01) |  |  | Ref | 1.46  (0.63, 3.40) | 1.53  (0.63, 3.71) | 1.39  (0.48, 3.97) |  |
| Diabetes |  |  |  |  | 0.776 |  |  |  |  |  | 0.428 |  |  |  |  |  | 0.374 |
| No | Ref | 1.01  (0.82, 1.23) | 1.13  (0.93, 1.38) | 1.46  (1.20, 1.77)^***^ |  |  | Ref | 0.94  (0.61, 1.45) | 0.82  (0.52, 1.30) | 1.23  (0.80, 1.90) |  |  | Ref | 1.36  (0.69, 2.67) | 1.33  (0.67, 2.62) | 1.54  (0.78, 3.05) |  |
| Yes | Ref | 1.10  (0.51, 2.37) | 1.28  (0.60, 2.70) | 1.35  (0.60, 3.06) |  |  | Ref | 0.21  (0.02, 1.81) | 0.45  (0.09, 2.37) | 1.28  (0.31, 5.38) |  |  | Ref | 0.30  (0.03, 3.19) | 0.00 (0.00, >99) | 3.58  (0.64, 19.90) |  |
| Hypertension |  |  |  |  | 0.591 |  |  |  |  |  | 0.923 |  |  |  |  |  | 0.789 |
| No | Ref | 0.76  (0.48, 1.22) | 0.94  (0.60, 1.47) | 1.26  (0.82, 1.94) |  |  | Ref | 0.98  (0.35, 2.76) | 0.49  (0.14, 1.66) | 0.95  (0.33, 2.69) |  |  | Ref | 2.13  (0.37, 12.24) | 0.98  (0.13, 7.51) | 1.02  (0.12, 8.73) |  |
| Yes | Ref | 1.08  (0.87, 1.34) | 1.18  (0.95, 1.45) | 1.49  (1.20, 1.84)^***^ |  |  | Ref | 0.83  (0.53, 1.32) | 0.83  (0.52, 1.33) | 1.29  (0.83, 2.01) |  |  | Ref | 1.01  (0.51, 2.01) | 1.04  (0.53, 2.06) | 1.57  (0.82, 2.99) |  |

Adjusted for sex, age, Townsend deprivation index, household income, physical activity, smoking status, alcohol status, cholesterol-lowering medication use, antihypertensive drugs use, insulin treatment, aspirin use, diabetes, and hypertension. HR: hazard ratio; CI: confidence interval; PUFAs: polyunsaturated fatty acids; DHA, docosahexaenoic acid; LA, linoleic acid; CVD, cardiovascular disease; IHD, ischemic heart disease.

**Supplementary Table 6**. Subgroup analysis between total PUFAs, omega-3, DHA, omega-6, LA, and omega-6/omega-3 ratio and all-cause, cardiovascular disease, and ischemic heart disease mortality in Cluster 3 (HR, 95%CI).

| **Subgroups** | **All-cause mortality** | | | | |  | **CVD mortality** | | | | |  | **IHD mortality** | | | | |
| --- | --- | --- | --- | --- | --- | --- | --- | --- | --- | --- | --- | --- | --- | --- | --- | --- | --- |
|  | **Q1** | **Q2** | **Q3** | **Q4** | ***P-int*** |  | **Q1** | **Q2** | **Q3** | **Q4** | ***P-int*** |  | **Q1** | **Q2** | **Q3** | **Q4** | ***P-int*** |
| **Total PUFAs** |  |  |  |  |  |  |  |  |  |  |  |  |  |  |  |  |  |
| Age (years) |  |  |  |  | 0.548 |  |  |  |  |  | 0.924 |  |  |  |  |  | 0.707 |
| <60 | Ref | 1.09  (0.86, 1.38) | 0.96  (0.67, 1.38) | 1.10  (0.67, 1.78) |  |  | Ref | 0.85  (0.54, 1.33) | 1.07  (0.57, 1.99) | 1.74  (0.86, 3.53) |  |  | Ref | 0.70  (0.40, 1.22) | 1.16  (0.57, 2.40) | 2.23  (1.04, 4.75)^*^ |  |
| ≥60 | Ref | 0.93  (0.83, 1.04) | 0.89  (0.77, 1.04) | 0.90  (0.70, 1.15) |  |  | Ref | 0.86  (0.68, 1.09) | 1.03  (0.77, 1.38) | 1.08  (0.67, 1.73) |  |  | Ref | 0.82  (0.62, 1.10) | 1.10  (0.78, 1.56) | 0.79  (0.40, 1.55) |  |
| Sex |  |  |  |  | 0.149 |  |  |  |  |  | 0.138 |  |  |  |  |  | 0.189 |
| Male | Ref | 0.93  (0.83, 1.04) | 0.93  (0.79, 1.09) | 1.02  (0.77, 1.34) |  |  | Ref | 0.84  (0.67, 1.06) | 1.14  (0.85, 1.53) | 1.60  (1.04, 2.46)^*^ |  |  | Ref | 0.78  (0.59, 1.03) | 1.18  (0.84, 1.65) | 1.54  (0.92, 2.56) |  |
| Female | Ref | 1.00  (0.79, 1.25) | 0.80  (0.61, 1.05) | 0.79  (0.54, 1.15) |  |  | Ref | 0.88  (0.54, 1.45) | 0.69  (0.38, 1.26) | 0.55  (0.21, 1.41) |  |  | Ref | 0.89  (0.46, 1.73) | 0.80  (0.37, 1.72) | 0.19  (0.03, 1.44) |  |
| Diabetes |  |  |  |  | 0.137 |  |  |  |  |  | 0.581 |  |  |  |  |  | 0.520 |
| No | Ref | 0.94  (0.83, 1.06) | 0.88  (0.75, 1.05) | 0.71  (0.53, 0.95)^*^ |  |  | Ref | 1.01  (0.78, 1.30) | 1.05  (0.75, 1.48) | 0.87  (0.48, 1.57) |  |  | Ref | 0.96  (0.70, 1.32) | 1.21  (0.81, 1.81) | 0.45  (0.17, 1.24) |  |
| Yes | Ref | 0.95  (0.79, 1.14) | 0.86  (0.67, 1.10) | 1.53  (1.09, 2.14)^*^ |  |  | Ref | 0.65  (0.45, 0.94)^*^ | 1.00  (0.66, 1.51) | 1.97  (1.16, 3.35)^*^ |  |  | Ref | 0.59  (0.38, 0.92)^*^ | 0.94  (0.57, 1.54) | 2.23  (1.23, 4.03)^**^ |  |
| Hypertension |  |  |  |  | 0.230 |  |  |  |  |  | 0.359 |  |  |  |  |  | 0.068 |
| No | Ref | 0.86  (0.63, 1.18) | 0.71  (0.45, 1.10) | 0.91  (0.46, 1.82) |  |  | Ref | 0.67  (0.35, 1.28) | 0.83  (0.38, 1.84) | 1.14  (0.39, 3.36) |  |  | Ref | 0.38  (0.15, 0.92)* | 0.86  (0.37, 2.01) | 0.63  (0.14, 2.87) |  |
| Yes | Ref | 0.95  (0.86, 1.06) | 0.91  (0.79, 1.06) | 0.93  (0.74, 1.18) |  |  | Ref | 0.90  (0.72, 1.12) | 1.08  (0.82, 1.43) | 1.25  (0.82, 1.91) |  |  | Ref | 0.90  (0.69, 1.17) | 1.16  (0.83, 1.62) | 1.22  (0.72, 2.08) |  |
| **Omega-3** |  |  |  |  |  |  |  |  |  |  |  |  |  |  |  |  |  |
| Age (years) |  |  |  |  | 0.269 |  |  |  |  |  | 0.463 |  |  |  |  |  | 0.494 |
| <60 | Ref | 0.82  (0.63, 1.06) | 0.78  (0.58, 1.06) | 0.90  (0.64, 1.26) |  |  | Ref | 0.63  (0.39, 1.03) | 0.80  (0.48, 1.34) | 0.86  (0.48, 1.54) |  |  | Ref | 0.51  (0.28, 0.92)^*^ | 0.82  (0.46, 1.48) | 0.81  (0.41, 1.59) |  |
| ≥60 | Ref | 0.83  (0.73, 0.93)^**^ | 0.76  (0.67, 0.87)^***^ | 0.75  (0.64, 0.88)^***^ |  |  | Ref | 0.77  (0.60, 0.97)^*^ | 0.67  (0.52, 0.88)^**^ | 0.76  (0.56, 1.02) |  |  | Ref | 0.83  (0.62, 1.10) | 0.61  (0.44, 0.86)^**^ | 0.75  (0.52, 1.09) |  |
| Sex |  |  |  |  | 0.543 |  |  |  |  |  | 0.704 |  |  |  |  |  | 0.844 |
| Male | Ref | 0.81  (0.71, 0.91)^**^ | 0.77  (0.68, 0.89)^***^ | 0.78  (0.67, 0.92)^**^ |  |  | Ref | 0.71  (0.56, 0.90)^**^ | 0.68  (0.52, 0.88)^**^ | 0.80  (0.59, 1.07) |  |  | Ref | 0.73  (0.55, 0.95)^*^ | 0.64  (0.47, 0.88)^**^ | 0.77  (0.54, 1.09) |  |
| Female | Ref | 0.82  (0.64, 1.06) | 0.67  (0.51, 0.87)^**^ | 0.67  (0.50, 0.91)^**^ |  |  | Ref | 0.87  (0.49, 1.52) | 0.75  (0.42, 1.34) | 0.71  (0.36, 1.39) |  |  | Ref | 1.02  (0.48, 2.16) | 0.75  (0.34, 1.66) | 0.76  (0.31, 1.89) |  |
| Diabetes |  |  |  |  | 0.162 |  |  |  |  |  | 0.875 |  |  |  |  |  | 0.878 |
| No | Ref | 0.78  (0.68, 0.89)^***^ | 0.75  (0.64, 0.87)^***^ | 0.69  (0.58, 0.83)^***^ |  |  | Ref | 0.86  (0.65, 1.13) | 0.73  (0.53, 1.00) | 0.78  (0.54, 1.12) |  |  | Ref | 0.99  (0.71, 1.38) | 0.77  (0.52, 1.14) | 0.74  (0.46, 1.17) |  |
| Yes | Ref | 0.87  (0.72, 1.05) | 0.76  (0.62, 0.94)^*^ | 0.85  (0.68, 1.08) |  |  | Ref | 0.56  (0.40, 0.79)^**^ | 0.64  (0.45, 0.92)^*^ | 0.73  (0.49, 1.09) |  |  | Ref | 0.50  (0.33, 0.75)^**^ | 0.51  (0.33, 0.80)^**^ | 0.74  (0.47, 1.17) |  |
| Hypertension |  |  |  |  | 0.832 |  |  |  |  |  | 0.520 |  |  |  |  |  | 0.814 |
| No | Ref | 0.75  (0.53, 1.06) | 0.83  (0.58, 1.20) | 0.71  (0.45, 1.12) |  |  | Ref | 0.68  (0.35, 1.31) | 1.00  (0.53, 1.91) | 0.59  (0.25, 1.41) |  |  | Ref | 0.58  (0.27, 1.23) | 0.67  (0.31, 1.45) | 0.37  (0.12, 1.12) |  |
| Yes | Ref | 0.82  (0.73, 0.92)^**^ | 0.74  (0.65, 0.84)^***^ | 0.76  (0.65, 0.88)^***^ |  |  | Ref | 0.75  (0.60, 0.94)^*^ | 0.66  (0.51, 0.85)^**^ | 0.79  (0.60, 1.05) |  |  | Ref | 0.78  (0.59, 1.02) | 0.64  (0.47, 0.88)^**^ | 0.82  (0.58, 1.15) |  |
| **DHA** |  |  |  |  |  |  |  |  |  |  |  |  |  |  |  |  |  |
| Age (years) |  |  |  |  | 0.055 |  |  |  |  |  | 0.053 |  |  |  |  |  | 0.098 |
| <60 | Ref | 0.89  (0.68, 1.15) | 0.95  (0.70, 1.30) | 1.06  (0.74, 1.52) |  |  | Ref | 1.07  (0.68, 1.67) | 0.89  (0.50, 1.60) | 1.43  (0.80, 2.56) |  |  | Ref | 1.04  (0.62, 1.74) | 0.86  (0.44, 1.70) | 1.30  (0.64, 2.63) |  |
| ≥60 | Ref | 0.82  (0.73, 0.92)^**^ | 0.76  (0.66, 0.87)^***^ | 0.73  (0.62, 0.86)^***^ |  |  | Ref | 0.75  (0.59, 0.94)^*^ | 0.67  (0.51, 0.88)^**^ | 0.70  (0.50, 0.97)^*^ |  |  | Ref | 0.71  (0.53, 0.94)^*^ | 0.58  (0.41, 0.82)^**^ | 0.70  (0.47, 1.04) |  |
| Sex |  |  |  |  | 0.288 |  |  |  |  |  | 0.522 |  |  |  |  |  | 0.851 |
| Male | Ref | 0.82  (0.72, 0.92)^**^ | 0.80  (0.70, 0.92)^**^ | 0.78  (0.66, 0.93)^**^ |  |  | Ref | 0.80  (0.64, 1.01) | 0.70  (0.53, 0.93)^*^ | 0.82  (0.60, 1.13) |  |  | Ref | 0.80  (0.61, 1.04) | 0.62  (0.44, 0.87)^**^ | 0.83  (0.58, 1.21) |  |
| Female | Ref | 0.78  (0.61, 0.99)^*^ | 0.68  (0.53, 0.89)^**^ | 0.63  (0.46, 0.87)^**^ |  |  | Ref | 0.73  (0.42, 1.24) | 0.73  (0.41, 1.30) | 0.69  (0.35, 1.38) |  |  | Ref | 0.54  (0.26, 1.13) | 0.62  (0.29, 1.32) | 0.52  (0.20, 1.35) |  |
| Diabetes |  |  |  |  | 0.866 |  |  |  |  |  | 0.247 |  |  |  |  |  | 0.575 |
| No | Ref | 0.83  (0.72, 0.95)^**^ | 0.80  (0.68, 0.93)^**^ | 0.72  (0.59, 0.87)^**^ |  |  | Ref | 0.83  (0.63, 1.09) | 0.81  (0.59, 1.12) | 0.92  (0.64, 1.32) |  |  | Ref | 0.91  (0.65, 1.27) | 0.72  (0.48, 1.08) | 0.95  (0.61, 1.48) |  |
| Yes | Ref | 0.77  (0.64, 0.93)^**^ | 0.72  (0.58, 0.89)^**^ | 0.78  (0.60, 1.00) |  |  | Ref | 0.74  (0.54, 1.02) | 0.55  (0.37, 0.83)^**^ | 0.62  (0.38, 0.99)^*^ |  |  | Ref | 0.60  (0.41, 0.89)^*^ | 0.51  (0.31, 0.83)^**^ | 0.59  (0.34, 1.03) |  |
| Hypertension |  |  |  |  | 0.689 |  |  |  |  |  | 0.340 |  |  |  |  |  | 0.818 |
| No | Ref | 0.79  (0.57, 1.10) | 0.66  (0.44, 0.98)^*^ | 0.85  (0.54, 1.34) |  |  | Ref | 0.66  (0.35, 1.23) | 0.57  (0.27, 1.22) | 0.85  (0.39, 1.85) |  |  | Ref | 0.53  (0.26, 1.08) | 0.35  (0.13, 0.94)^*^ | 0.52  (0.19, 1.40) |  |
| Yes | Ref | 0.82  (0.73, 0.91)^***^ | 0.78  (0.69, 0.89)^***^ | 0.73  (0.62, 0.86)^***^ |  |  | Ref | 0.82  (0.65, 1.02) | 0.73  (0.56, 0.95)^*^ | 0.78  (0.57, 1.06) |  |  | Ref | 0.80  (0.61, 1.04) | 0.68  (0.49, 0.94)^*^ | 0.83  (0.57, 1.20) |  |
| **Omega-6** |  |  |  |  |  |  |  |  |  |  |  |  |  |  |  |  |  |
| Age (years) |  |  |  |  | 0.772 |  |  |  |  |  | 0.880 |  |  |  |  |  | 0.849 |
| <60 | Ref | 1.23  (0.98, 1.56) | 0.96  (0.68, 1.35) | 0.91  (0.49, 1.68) |  |  | Ref | 0.87  (0.55, 1.37) | 1.41  (0.82, 2.43) | 1.30  (0.52, 3.28) |  |  | Ref | 0.72  (0.41, 1.28) | 1.64  (0.90, 3.01) | 1.57  (0.56, 4.42) |  |
| ≥60 | Ref | 0.93  (0.83, 1.04) | 0.87  (0.74, 1.02) | 0.92  (0.71, 1.20) |  |  | Ref | 0.84  (0.66, 1.06) | 1.17  (0.87, 1.56) | 1.05  (0.62, 1.79) |  |  | Ref | 0.83  (0.62, 1.11) | 1.14  (0.79, 1.64) | 0.99  (0.50, 1.95) |  |
| Sex |  |  |  |  | 0.124 |  |  |  |  |  | 0.057 |  |  |  |  |  | 0.104 |
| Male | Ref | 0.97  (0.87, 1.09) | 1.00  (0.85, 1.18) | 0.87  (0.63, 1.20) |  |  | Ref | 0.89  (0.71, 1.12) | 1.40  (1.06, 1.86)^*^ | 1.46  (0.88, 2.42) |  |  | Ref | 0.85  (0.65, 1.12) | 1.38  (0.99, 1.93) | 1.42  (0.77, 2.62) |  |
| Female | Ref | 0.93  (0.75, 1.17) | 0.64  (0.48, 0.85)^**^ | 0.89  (0.61, 1.30) |  |  | Ref | 0.57  (0.34, 0.96)^*^ | 0.65  (0.36, 1.16) | 0.44  (0.15, 1.23) |  |  | Ref | 0.55  (0.27, 1.12) | 0.69  (0.31, 1.50) | 0.39  (0.09, 1.68) |  |
| Diabetes |  |  |  |  | 0.216 |  |  |  |  |  | 0.560 |  |  |  |  |  | 0.489 |
| No | Ref | 0.95  (0.84, 1.07) | 0.83  (0.70, 0.99)^*^ | 0.80  (0.59, 1.07) |  |  | Ref | 0.87  (0.67, 1.13) | 1.08  (0.77, 1.51) | 0.72  (0.37, 1.43) |  |  | Ref | 0.83  (0.60, 1.15) | 1.15  (0.77, 1.73) | 0.52  (0.19, 1.41) |  |
| Yes | Ref | 1.01  (0.84, 1.21) | 0.99  (0.77, 1.28) | 1.17  (0.77, 1.78) |  |  | Ref. | 0.78  (0.55, 1.11) | 1.50  (1.01, 2.24)^*^ | 1.94  (1.04, 3.64)^*^ |  |  | Ref. | 0.77  (0.50, 1.17) | 1.42  (0.88, 2.29) | 2.27  (1.13, 4.56)^*^ |  |
| Hypertension |  |  |  |  | 0.131 |  |  |  |  |  | 0.190 |  |  |  |  |  | **0.036** |
| No | Ref | 0.76  (0.55, 1.06) | 0.75  (0.49, 1.15) | 0.64  (0.28, 1.49) |  |  | Ref | 0.58  (0.30, 1.12) | 0.79  (0.36, 1.74) | 1.19  (0.35, 4.03) |  |  | Ref | 0.29  (0.11, 0.75)^*^ | 0.84  (0.36, 1.97) | 0.43  (0.06, 3.25) |  |
| Yes | Ref | 1.00  (0.90, 1.11) | 0.90  (0.77, 1.05) | 0.95  (0.74, 1.23) |  |  | Ref | 0.90  (0.72, 1.12) | 1.30  (0.99, 1.70) | 1.14  (0.70, 1.88) |  |  | Ref | 0.93  (0.71, 1.22) | 1.33  (0.95, 1.85) | 1.32  (0.73, 2.38) |  |
| **LA** |  |  |  |  |  |  |  |  |  |  |  |  |  |  |  |  |  |
| Age (years) |  |  |  |  | 0.847 |  |  |  |  |  | 0.896 |  |  |  |  |  | 0.939 |
| <60 | Ref | 1.31  (1.04, 1.65)^*^ | 0.81  (0.56, 1.19) | 1.15  (0.69, 1.93) |  |  | Ref | 1.10  (0.72, 1.69) | 0.94  (0.49, 1.79) | 1.90  (0.90, 4.03) |  |  | Ref | 0.90  (0.53, 1.53) | 1.10  (0.53, 2.25) | 2.48  (1.10, 5.58)^*^ |  |
| ≥60 | Ref | 0.97  (0.86, 1.08) | 0.90  (0.77, 1.06) | 0.96  (0.74, 1.24) |  |  | Ref | 0.89  (0.70, 1.12) | 1.16  (0.86, 1.55) | 0.99  (0.58, 1.68) |  |  | Ref | 0.93  (0.71, 1.24) | 1.07  (0.74, 1.56) | 1.04  (0.55, 1.99) |  |
| Sex |  |  |  |  | 0.143 |  |  |  |  |  | 0.102 |  |  |  |  |  | 0.123 |
| Male | Ref | 1.04  (0.93, 1.16) | 0.97  (0.82, 1.14) | 1.01  (0.76, 1.35) |  |  | Ref | 0.97  (0.77, 1.21) | 1.30  (0.97, 1.73) | 1.41  (0.87, 2.29) |  |  | Ref | 0.94  (0.72, 1.23) | 1.24  (0.87, 1.75) | 1.57  (0.91, 2.73) |  |
| Female | Ref | 0.93  (0.74, 1.16) | 0.65  (0.48, 0.87)^**^ | 0.86  (0.58, 1.28) |  |  | Ref | 0.69  (0.42, 1.13) | 0.54  (0.28, 1.05) | 0.61  (0.24, 1.58) |  |  | Ref | 0.80  (0.42, 1.54) | 0.45  (0.17, 1.20) | 0.68  (0.20, 2.36) |  |
| Diabetes |  |  |  |  | 0.091 |  |  |  |  |  | 0.439 |  |  |  |  |  | 0.370 |
| No | Ref | 0.99  (0.87, 1.12) | 0.84  (0.71, 1.00) | 0.83  (0.62, 1.10) |  |  | Ref | 0.93  (0.72, 1.21) | 1.02  (0.73, 1.44) | 0.76  (0.40, 1.45) |  |  | Ref | 0.89  (0.64, 1.23) | 1.04  (0.68, 1.59) | 0.75  (0.33, 1.71) |  |
| Yes | Ref | 1.09  (0.92, 1.30) | 0.94  (0.73, 1.23) | 1.43  (0.97, 2.09) |  |  | Ref. | 0.92  (0.66, 1.28) | 1.28  (0.83, 1.96) | 2.27  (1.26, 4.07)^**^ |  |  | Ref. | 0.98  (0.67, 1.44) | 1.11  (0.65, 1.89) | 2.74  (1.44, 5.23)^**^ |  |
| Hypertension |  |  |  |  | 0.126 |  |  |  |  |  | 0.164 |  |  |  |  |  | **0.031** |
| No | Ref | 0.84  (0.61, 1.16) | 0.78  (0.51, 1.19) | 0.46  (0.20, 1.07) |  |  | Ref | 0.73  (0.39, 1.37) | 0.86  (0.39, 1.89) | 0.58  (0.14, 2.50) |  |  | Ref | 0.40  (0.17, 0.93)^*^ | 0.75  (0.30, 1.84) | 0.34  (0.04, 2.61) |  |
| Yes | Ref | 1.05  (0.94, 1.17) | 0.89  (0.76, 1.04) | 1.08  (0.85, 1.37) |  |  | Ref | 0.97  (0.78, 1.21) | 1.17  (0.88, 1.55) | 1.34  (0.86, 2.11) |  |  | Ref | 1.06  (0.81, 1.37) | 1.15  (0.80, 1.64) | 1.68  (1.00, 2.82) |  |
| **Omega-6/3** |  |  |  |  |  |  |  |  |  |  |  |  |  |  |  |  |  |
| Age (years) |  |  |  |  | 0.403 |  |  |  |  |  | 0.954 |  |  |  |  |  | 0.719 |
| <60 | Ref | 1.20  (0.87, 1.65) | 1.07  (0.77, 1.47) | 1.26  (0.92, 1.71) |  |  | Ref | 0.69  (0.38, 1.23) | 0.96  (0.57, 1.61) | 1.08  (0.64, 1.79) |  |  | Ref | 0.71  (0.36, 1.41) | 1.04  (0.56, 1.91) | 1.19  (0.65, 2.16) |  |
| ≥60 | Ref | 1.11  (0.96, 1.27) | 1.14  (0.99, 1.32) | 1.40  (1.22, 1.61)^***^ |  |  | Ref | 1.19  (0.90, 1.57) | 1.17  (0.88, 1.56) | 1.44  (1.08, 1.91)^*^ |  |  | Ref | 1.21  (0.86, 1.71) | 1.25  (0.89, 1.77) | 1.59  (1.13, 2.25)^**^ |  |
| Sex |  |  |  |  | 0.027 |  |  |  |  |  | 0.767 |  |  |  |  |  | 0.667 |
| Male | Ref | 1.15  (1.00, 1.34) | 1.15  (0.99, 1.33) | 1.42  (1.23, 1.64)^***^ |  |  | Ref | 1.14  (0.86, 1.50) | 1.17  (0.88, 1.53) | 1.45  (1.11, 1.90)^**^ |  |  | Ref | 1.17  (0.84, 1.62) | 1.20  (0.86, 1.67) | 1.58  (1.14, 2.17)^**^ |  |
| Female | Ref | 1.07  (0.82, 1.39) | 1.10  (0.83, 1.46) | 1.40  (1.05, 1.87)^*^ |  |  | Ref | 0.88  (0.50, 1.56) | 1.00  (0.55, 1.79) | 1.00  (0.52, 1.91) |  |  | Ref | 0.83  (0.37, 1.88) | 1.41  (0.66, 3.01) | 1.07  (0.44, 2.61) |  |
| Diabetes |  |  |  |  | 0.289 |  |  |  |  |  | 0.996 |  |  |  |  |  | 0.918 |
| No | Ref | 1.17  (1.00, 1.38) | 1.17  (0.99, 1.38) | 1.47  (1.25, 1.73)^***^ |  |  | Ref | 1.23  (0.88, 1.71) | 1.14  (0.81, 1.59) | 1.34  (0.96, 1.88) |  |  | Ref | 1.32  (0.88, 1.98) | 1.26  (0.83, 1.90) | 1.38  (0.91, 2.10) |  |
| Yes | Ref | 1.07  (0.87, 1.31) | 1.10  (0.89, 1.36) | 1.33  (1.08, 1.65)^**^ |  |  | Ref | 0.89  (0.61, 1.30) | 1.18 (0.81, 1.71) | 1.44  (1.00, 2.08) |  |  | Ref | 0.85  (0.53, 1.35) | 1.22  (0.78, 1.90) | 1.72  (1.12, 2.64)^*^ |  |
| Hypertension |  |  |  |  | 0.817 |  |  |  |  |  | 0.289 |  |  |  |  |  | 0.347 |
| No | Ref | 1.18  (0.78, 1.79) | 1.29  (0.86, 1.92) | 1.21  (0.80, 1.83) |  |  | Ref | 1.61  (0.78, 3.31) | 1.39  (0.66, 2.90) | 1.13  (0.52, 2.50) |  |  | Ref | 1.84  (0.75, 4.51) | 2.05  (0.85, 4.95) | 1.44  (0.54, 3.83) |  |
| Yes | Ref | 1.14  (0.99, 1.30) | 1.13  (0.98, 1.29) | 1.45  (1.26, 1.66)^***^ |  |  | Ref | 1.04  (0.80, 1.35) | 1.13  (0.86, 1.47) | 1.41  (1.08, 1.83)^*^ |  |  | Ref | 1.04  (0.75, 1.44) | 1.15  (0.83, 1.59) | 1.54  (1.12, 2.11)^**^ |  |

Adjusted for sex, age, Townsend deprivation index, household income, physical activity, smoking status, alcohol status, cholesterol-lowering medication use, antihypertensive drugs use, insulin treatment, aspirin use, diabetes, and hypertension. HR: hazard ratio; CI: confidence interval; PUFAs: polyunsaturated fatty acids; DHA, docosahexaenoic acid; LA, linoleic acid; CVD, cardiovascular disease; IHD, ischemic heart disease.


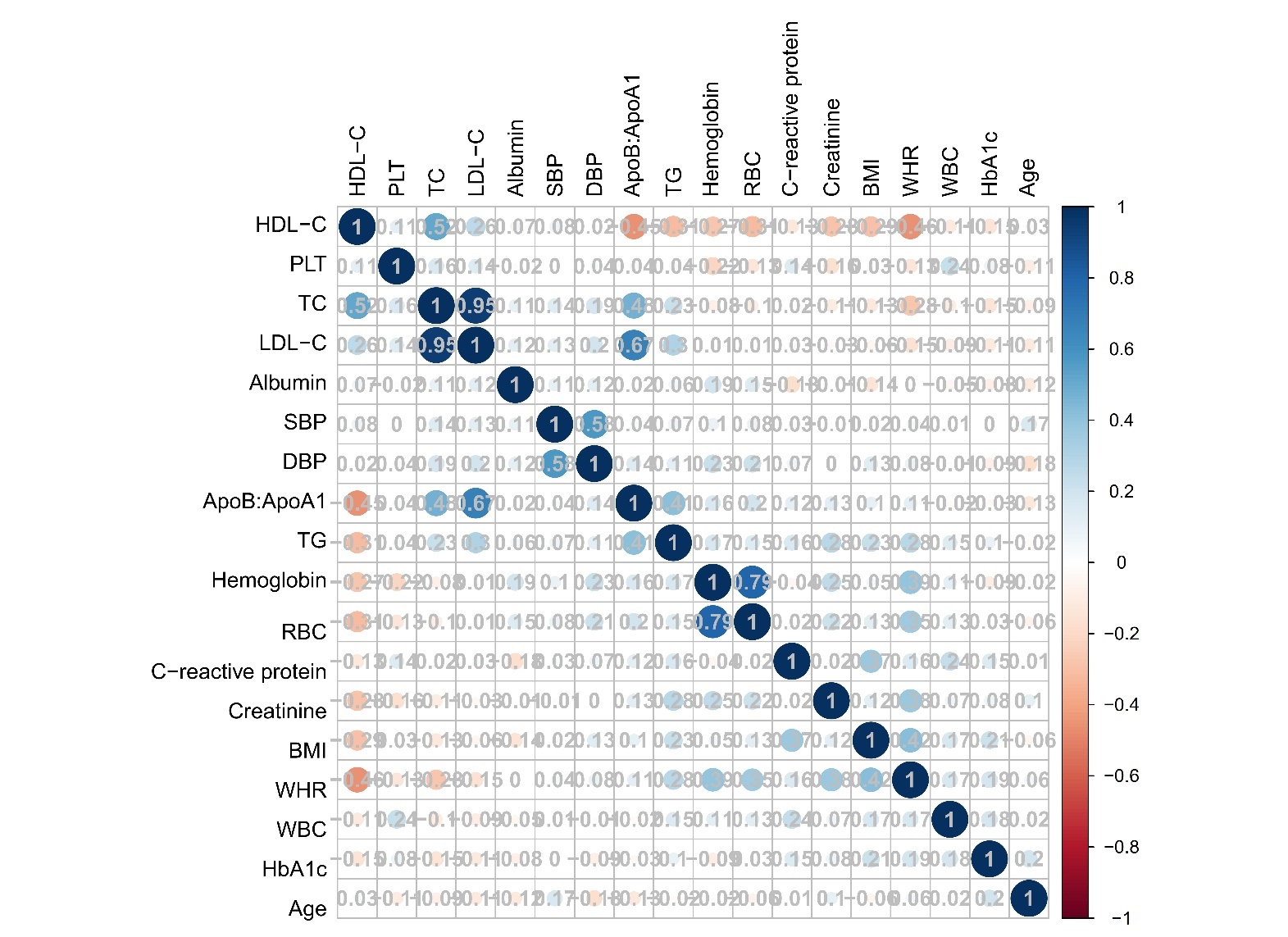


Supplementary Figure 1. Linear correlation between all pairs of features (Pearson correlation). BMI: body mass index; WHR: waist-hip ratio; DBP: diastolic blood pressure; SBP: systolic blood pressure; TC: total cholesterol; TG: total triglycerides; HDL-C: high-density lipoprotein-cholesterol; ApoB: ApoA1: apolipoprotein B: apolipoproteinA1; HbA1c: glycated hemoglobin; WBC: white blood cell count; PLT: platelet count.


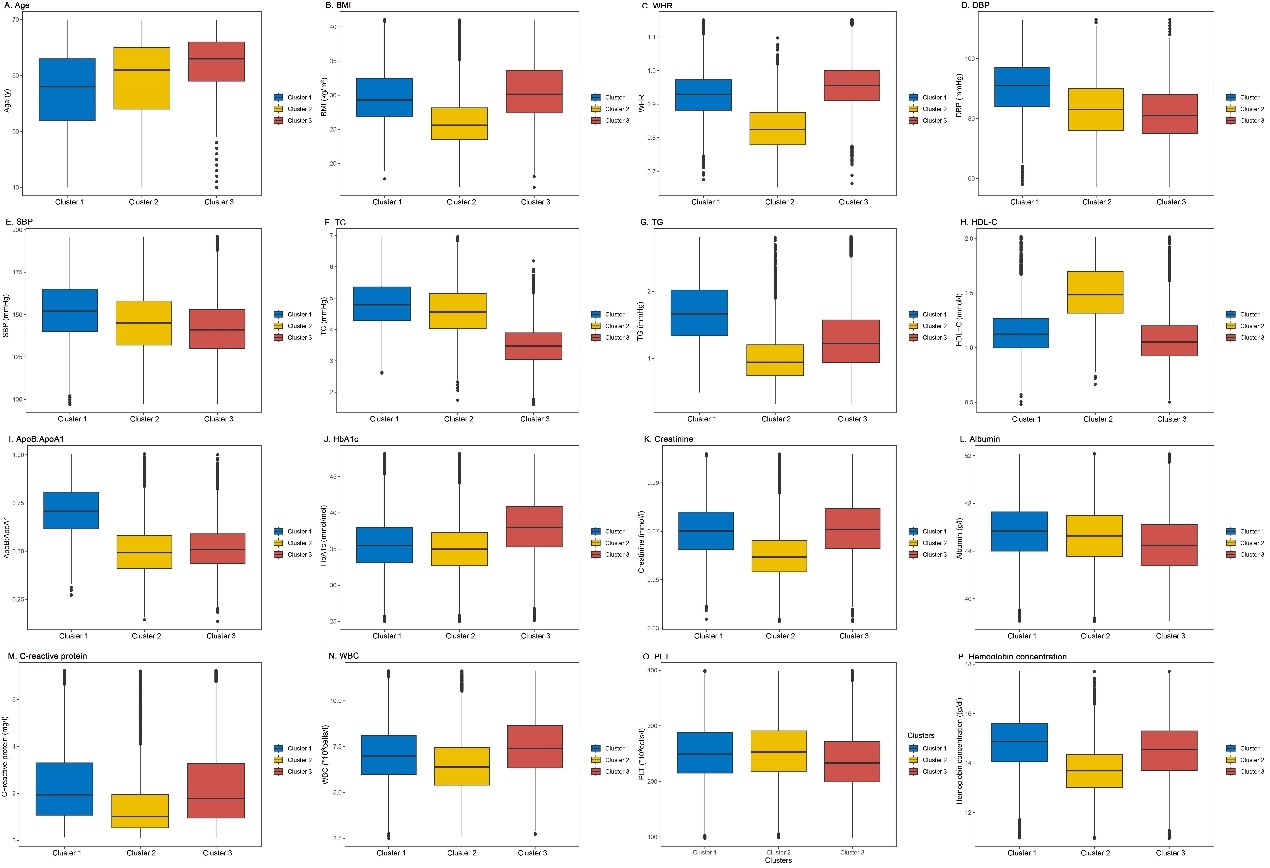


**Supplementary Figure 2.** The box diagram of clustering indicators. BMI: body mass index; WHR: waist-hip ratio; DBP: diastolic blood pressure; SBP: systolic blood pressure; TC: total cholesterol; TG: total triglycerides; HDL-C: high-density lipoprotein-cholesterol; ApoB: ApoA1: apolipoprotein B: apolipoproteinA1; HbA1c: glycated hemoglobin; WBC: white blood cell count; PLT: platelet count.


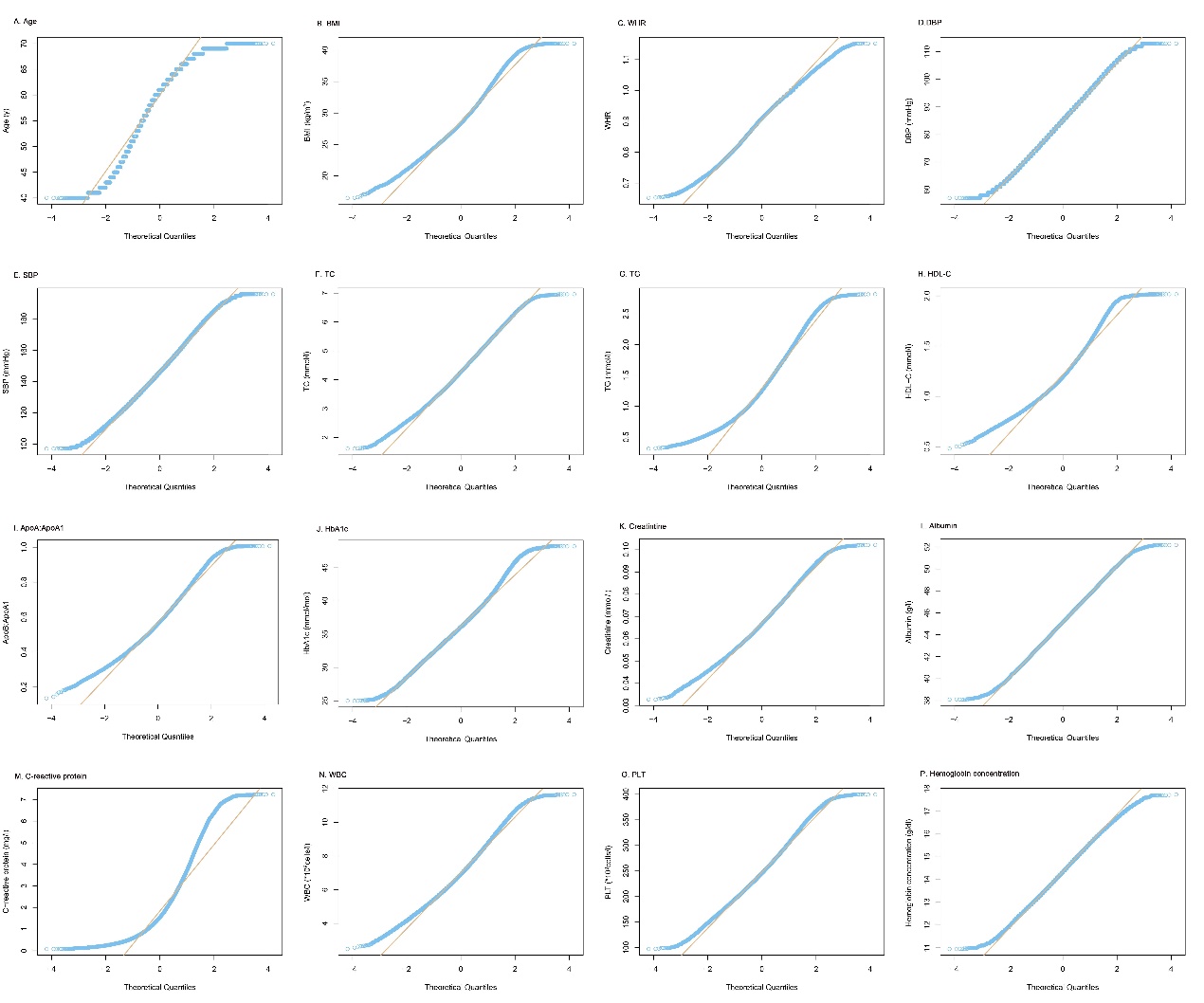


**Supplementary Figure 3.** The distribution patterns of clustering variables were evaluated by creating Q-Q plots. BMI: body mass index; WHR: waist-hip ratio; DBP: diastolic blood pressure; SBP: systolic blood pressure; TC: total cholesterol; TG: total triglycerides; HDL-C: high-density lipoprotein-cholesterol; ApoB: ApoA1: apolipoprotein B: apolipoproteinA1; HbA1c: glycated hemoglobin; WBC: white blood cell count; PLT: platelet count.


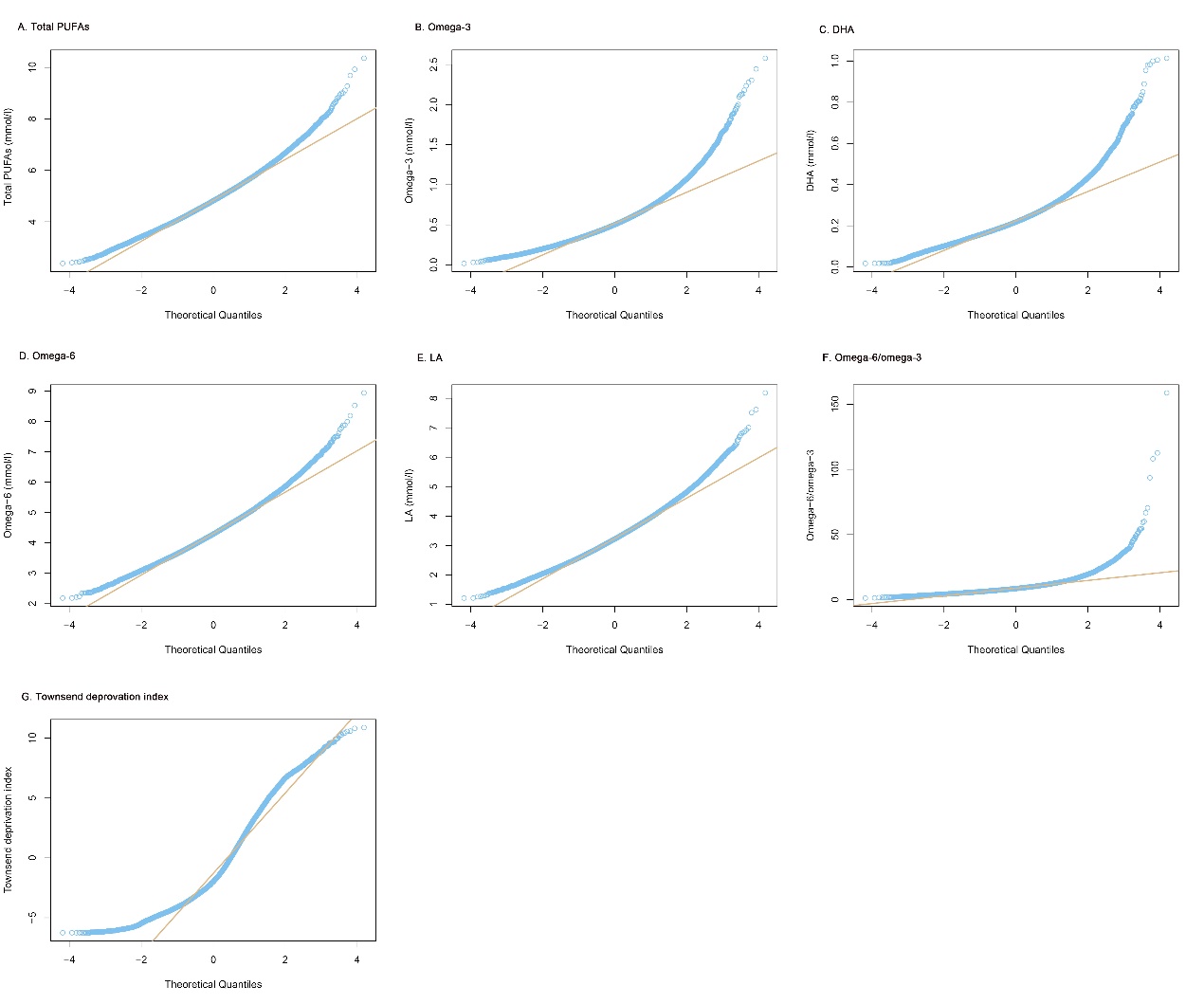


**Supplementary Figure 4.** The distribution patterns of continuous variables were evaluated by creating Q-Q plots. PUFAs: polyunsaturated fatty acids; DHA, docosahexaenoic acid; LA, linoleic acid.
